# Mukaiyama Aldol Reactions in Aqueous Media

**DOI:** 10.1002/adsc.201300798

**Published:** 2013-10-31

**Authors:** Taku Kitanosono, Shū Kobayashi

**Affiliations:** a Department of Chemistry, School of Science, The University of TokyoHongo, Bunkyo-ku, Tokyo, Japan, Fax: (+81)-(0)3-5684-0634; phone: (+81)-(0)3-5841-4790 e-mail: shu_kobayashi@chem.s.u-tokyo.ac.jp

**Keywords:** aldol reaction, asymmetric catalysis, green chemistry, Lewis acids, water

## Abstract

Mukaiyama aldol reactions in aqueous media have been surveyed. While the original Mukaiyama aldol reactions entailed stoichiometric use of Lewis acids in organic solvents under strictly anhydrous conditions, Mukaiyama aldol reactions in aqueous media are not only suitable for green sustainable chemistry but are found to produce singular phenomena. These findings led to the discovery of a series of water-compatible Lewis acids such as lanthanide triflates in 1991. Our understanding on these beneficial effects in the presence of water will be deepened through the brilliant examples collected in this review.

1 Introduction

2 Rate Enhancement by Water in the Mukaiyama Aldol Reaction

3 Lewis Acid Catalysis in Aqueous or Organic Solvents

3.1 Water-Compatible Lewis Acids

4 Lewis-Base Catalysis in Aqueous or Organic Solvents

5 The Mukaiyama Aldol Reactions in 100% Water

6 Asymmetric Catalysts in Aqueous Media and Water

7 Conclusions and Perspective

*Shū Kobayashi* studied at the University of Tokyo, receiving his Ph.D. in 1988 working under the direction of Professor T. Mukaiyama. Following an initial period as assistant professor, he was promoted to lecturer then associate professor at Science University of Tokyo (SUT). In 1998, he moved to the Graduate School of Pharmaceutical Sciences, the University of Tokyo, as full professor. In 2007, he was appointed to his current position as professor of organic chemistry in the Department of Chemistry, Faculty of Science, the University of Tokyo. He has held various visiting professorships, including the Université Louis Pasteur, Strasbourg (1993), Kyoto University (1995), Nijmegen University (1996), Philipps-University of Marburg (1997), Paris-Sud (2010). Professor Kobayashi has wide-ranging research interests that include the development of new synthetic methods and novel catalysts, organic reactions in water, solid-phase synthesis, total synthesis of biologically interesting compounds, and organometallic chemistry. He has held numerous named lectureships and is a recipient of many prestigious awards, including the Chemical Society of Japan Award for Young Chemists (1991), Ciba-Geigy Research Foundation Award (1994), Springer Award in Organometallic Chemistry (1997), IBM Science Award (2001), Organic Reactions Lecturer (2002), Nagoya Silver Medal (2002), Mitsui Chemical Catalysis Science Award (2005), JSPS Prize (2005), the Arthur C. Cope Scholar Award from the American Chemical Society (2006), Howard Memorial Lecturer (2006), C.S. Hamilton Award (2007), Merck-Cambridge Lecturer (2007), Humboldt Research Award (2013), and Green Chemistry Minister of Education Award (2013). Professor Kobayashi is a member of the Editorial Board of *Advanced Synthesis & Catalysis*.

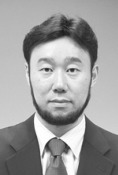

*Taku Kitanosono* earned his B.Sc. (2010) and M.Sc. (2012) degrees from the University of Tokyo under the supervision of Professor Shū Kobayashi. He is now a graduate student of the Ph.D. course of the University of Tokyo and is also a junior research fellow of the Japan Society for the Promotion of Science (JSPS). He got the Student Poster Award – First Prize at the 3rd Asia-Oceania Conference on Green & Sustainable Chemistry (2011). His research interests include the development of new synthetic methods and novel catalyses in aqueous environments.

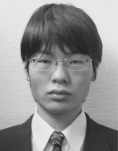

## 1 Introduction

The reactions of silicon enolates with aldehydes, renowned as Mukaiyama aldol reactions, are among the most reliable carbon-carbon bond-forming reactions in organic synthesis.^1^ Silicon enolates are sometimes called silyl enol ethers (silicon enolates derived from ketones) and ketene silyl acetals (silicon enolates derived from esters). They react with aldehydes in the presence of Lewis acids or fluoride anions to afford aldol adducts. Classical aldol reactions under basic conditions suffer from generally low selectivity due to their reversibility and many competitive side pathways such as dehydration, dimerization, polymerization and self-condensation,^2^ Mukaiyama aldol reactions proceed under acidic conditions and can avoid these competitive processes.

Since the first report on the Mukaiyama aldol reactions mediated by a stoichiometric amount of TiCl_4_ in 1973, many other stoichiometric Lewis acids such as BF_3_, SnCl_4_, AlCl_3_, FeCl_3_, and ZnCl_2_ have been developed.^3^ Furthermore, many other electrophiles such as ketones,^4^ acetals,1b,^5^ thioacetals,^6^ imines,^7^ and ketimines,^8^ have also been applied to the system. Even for nucleophiles, silicon dienolates,^9^ allylsilanes,^10^ and silyl cyanides,^11^ as well as other materials have been exploited under acidic conditions. The amounts of Lewis acids have decreased from stoichiometric to catalytic levels. The aldol-type reactions of acetals with silicon enolates proceeded smoothly using a catalytic amount of Me_3_SiOTf.^12^ Triphenylmethyl (trityl, Tr) salts were found to work as non-metal Lewis acids and were applied to the Mukaiyama aldol reaction.^13^ In the presence of a catalytic amount of TrClO_4_ or TrSbCl_6_, the aldol reactions of silicon enolates with aldehydes or acetals proceeded smoothly to afford the corresponding adducts in high yields. Another important aspect of silicon enolates and the Mukaiyama aldol reaction in organic chemistry and organic synthesis is the development of chiral Lewis acids. After the discovery of the Mukaiyama aldol reactions and in light of the increasing demand for asymmetric reactions, many chiral Lewis acids have been developed for the Mukaiyama aldol reaction. Chiral Lewis acids act as catalysts, and thus catalytic asymmetric aldol reactions have been attained.

In all cases, the reactions were carried out in organic solvents and mostly under strictly anhydrous conditions. On the other hand, organic reactions in water have been of great interest because water is a safe, inexpensive, and clean solvent. However, for the Mukaiyama aldol reaction, the use of water as a solvent was believed to be impossible because (i) Lewis acids as activators or catalysts were believed to be incompatible with water because of the weakness of the non-covalent interactions and (ii) most silicon enolates decompose in the presence of water.

However, efforts to develop the Mukaiyama aldol reaction in aqueous media led to many fruitful scientific advances not only in organic chemistry and organic synthesis but also in inorganic chemistry and biochemistry. In this article, the development of the Mukaiyama aldol reaction in aqueous media is surveyed.

## 2 Rate Enhancement by Water in the Mukaiyama Aldol Reaction

In 1986, Lubineau et al. reported that benzaldehyde **1** could react with silicon enolate **2** derived from cyclohexanone in water without addition of any Lewis acids or fluoride anions.^14^ While the same reaction carried out in the presence of a stoichiometric amount of TiCl_4_ in dichloromethane (DCM) proceeded rapidly (82% yield after 2 h), the reaction in water proceeded more slowly (43% yield after 5 d). The diastereoselectivity of the product was reversed in comparison with the conventional TiCl_4_-catalyzed reaction (Table 1).^15^ Although the reaction proceeded rather slowly, an obvious rate acceleration was observed in water because no reaction occurred in DCM without any Lewis acids.

**Table 1:** Water-accelerated Mukaiyama aldol reaction

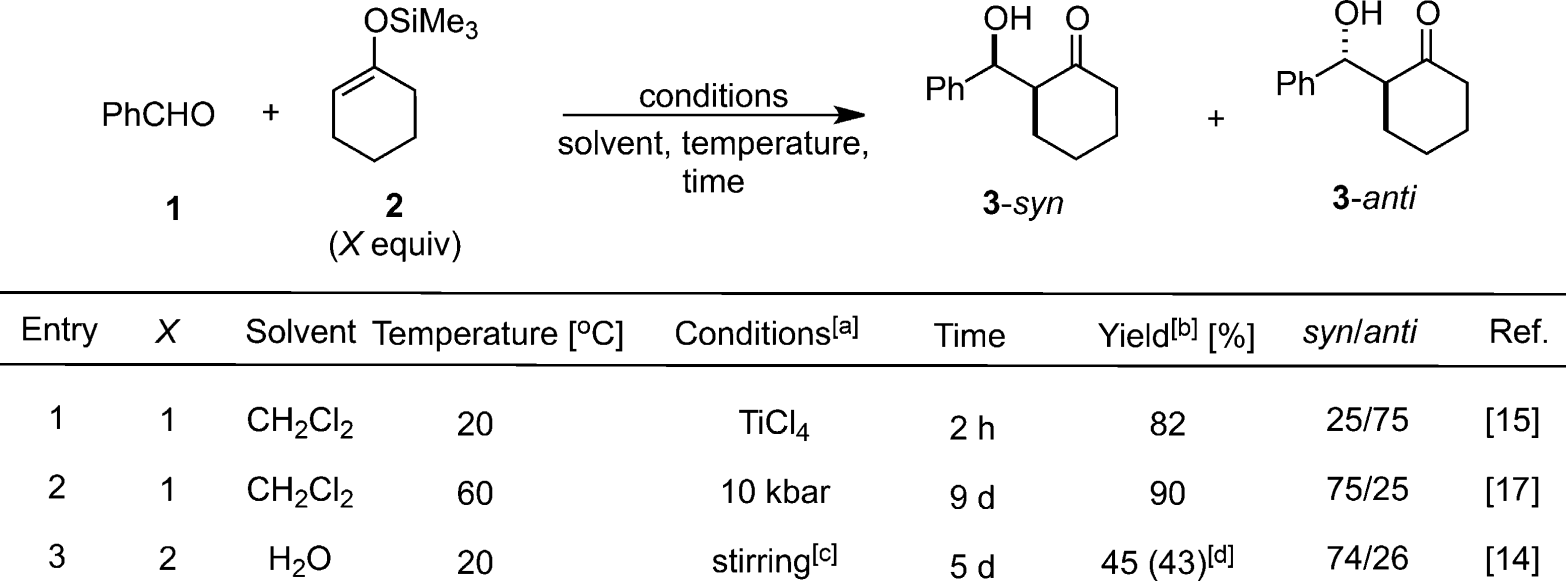

^[a]^ All reactions were carried out under atmospheric pressure at a concentration of 0.4 M of the limiting component.

^[b]^ Determined by NMR analysis.

^[c]^ Vigorous magnetic stirring or violent shaking.

^[d]^ Isolated yield.

Interestingly, the rate acceleration and the stereochemical outcome in water bear a striking resemblance to the reaction performed under high pressure, as shown in Table 1. A curious coincidence between reactions in water and those under high pressure has been observed ever since.^16^ Yamamoto et al. indicated that the transition state for the *syn*-selective pathway under high pressure was more compact.^17^ As is often the case for reactions that possess a smaller molar volume of a transition state, a key factor of the rate acceleration in water is the entropy-driven aggregation as a result of hydrophobic interactions. It is considered that these attractive outcomes arise from the unique properties of water, such as its high dielectric constant and high cohesive energy density (estimated 22 kbar) relative to conventional organic solvents.^18^,^19^ In addition, it is noted that water can function as a Lewis base (see Section 3) toward silicon enolates to afford the resulting silicate, which is more nucleophilic than the original silicon enolate.

**Scheme 1:**
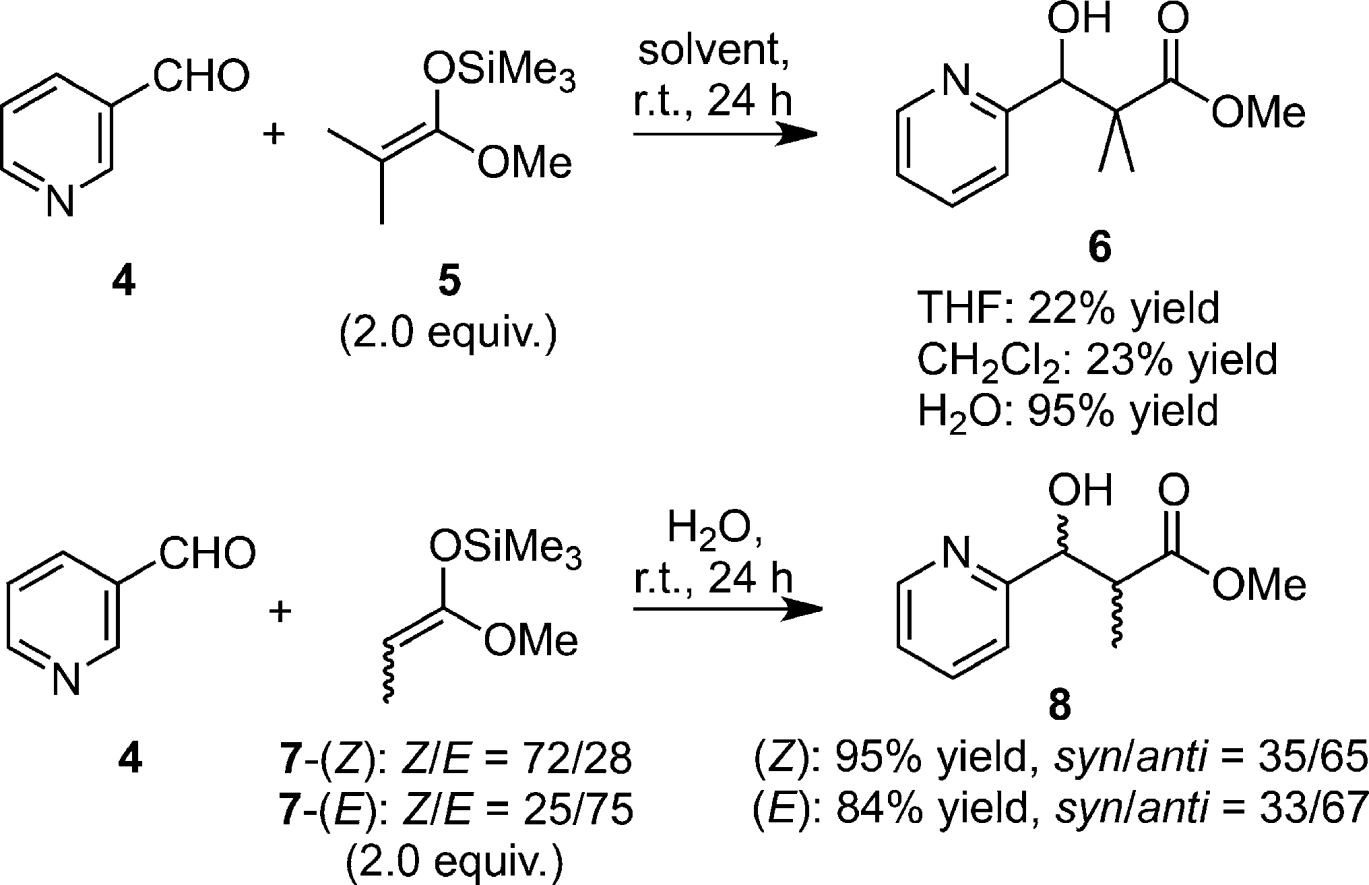
Water-accelerated Mukaiyama aldol reactions of ketene silyl acetals.

Similar rate acceleration was observed in the Mukaiyama aldol reaction using ketene silyl acetals. Their reaction with 2-pyridinecarboxyaldehyde **4** in water was found to be approximately four times faster than that in organic solvents (Scheme 1).^20^ The reaction exhibited *anti*-selectivity in water, irrespective of the *E*/*Z* ratio of starting enolate **7**.

**Scheme 2:**
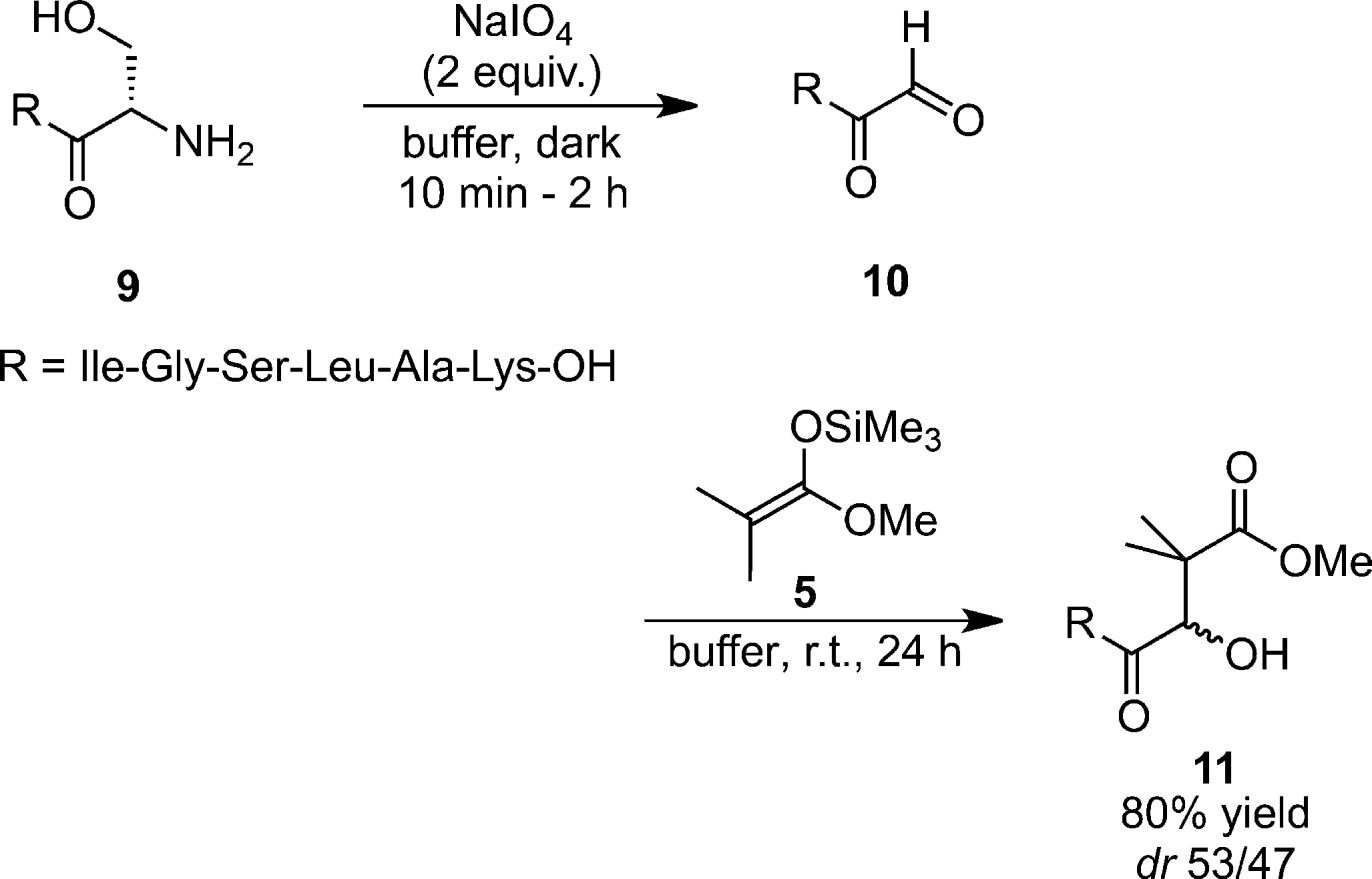
Mukaiyama aldol reactions of a peptide aldehyde.

The scope of the catalyst-free, water-based Mukaiyama aldol reaction was explored through its application to the site-selective functionalization of N-terminal aldehydes of peptides and proteins. Vigorous stirring of a dipeptide aldehyde generated by periodate oxidation from a polypeptide with a ketene silyl acetal in 10 mM sodium phosphate buffer (pH 7.0) was also discovered to lead to the aldol product with high efficiency (Scheme 2).^21^ The Mukaiyama aldol reaction could be applied to various N-terminal aldehydes of peptides and proteins bearing a wide range of functional groups under very mild conditions. As shown in the case of myoglobin, the reaction could be carried out without disturbing either the tertiary structure or the enzymatic activity of the protein.

**Scheme 3:**
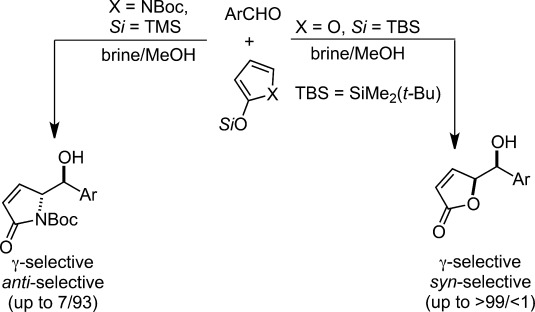
Diastereoselective vinylogous Mukaiyama aldol reactions in aqueous media: pyrrole *vs.* furan 2-silyloxy dienes.

The dual role of water as a reaction medium and promoter was also highlighted in vinylogous Mukaiyama aldol reactions of pyrrole and furan 2-silyloxy dienes in aqueous media.^22^ *N*-Boc-2-(trimethylsilyloxy)pyrrole reacted with benzaldehyde in aqueous media to afford the desired product with almost complete γ-site selectivity and good diastereomeric ratio in favor of the *anti*-configured isomer, while the reaction failed in organic solvents or under neat conditions. A remarkable switch of diastereoselectivity was observed when furan 2-siloxydiene was used (Scheme 3).

**Scheme 4:**
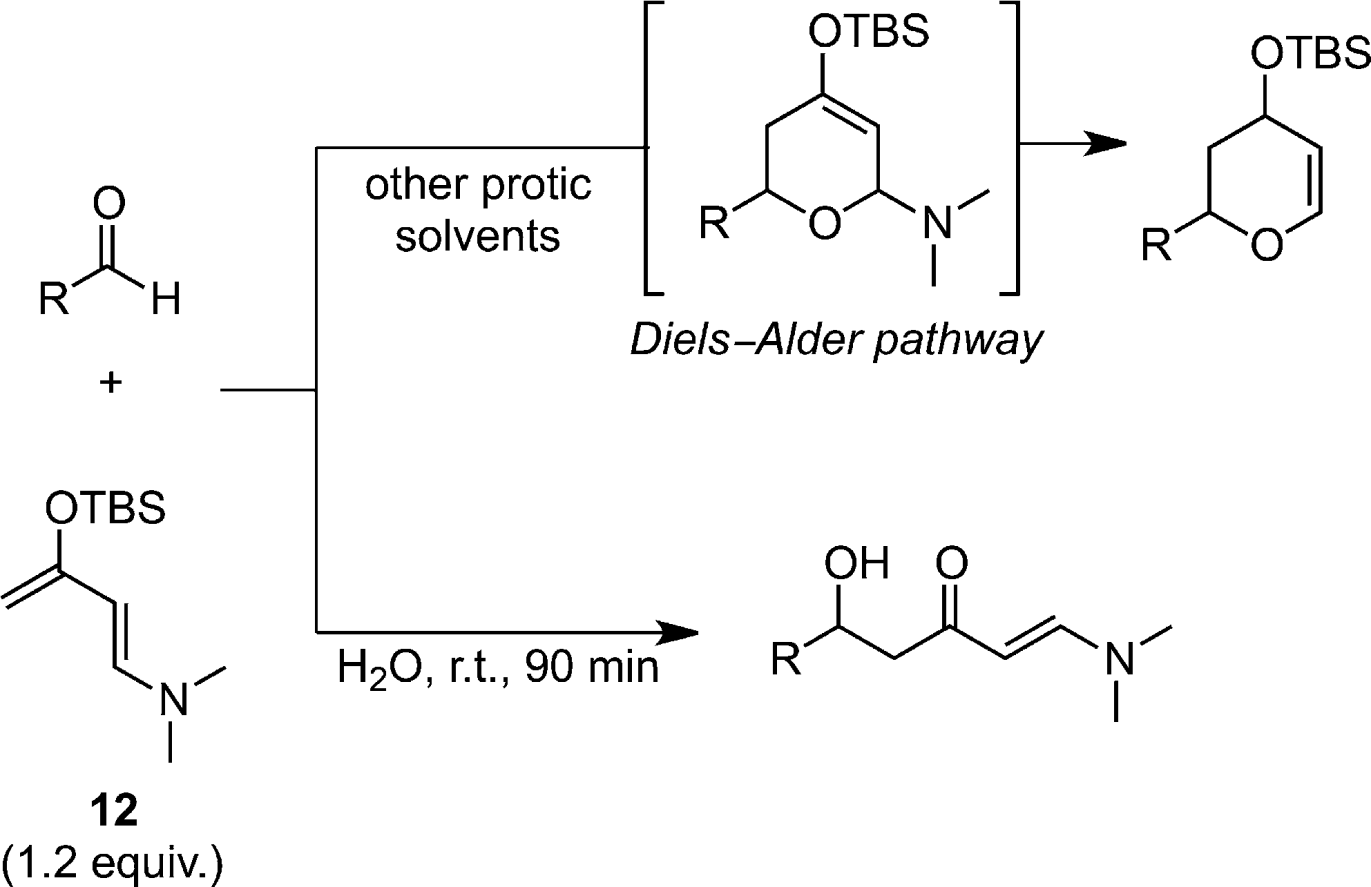
Reaction of Rawal’s diene **12** in water.

The Mukaiyama aldol reaction of electron-rich Rawal’s diene **12** with carbonyl compounds proceeded without any activator in pure water (Scheme 4).^23^ ^1^H NMR analyses indicated that the solvents influenced the reaction pathway and the aldol adducts were obtained in water, while the cycloadduct was obtained through the Diels–Alder pathway in other protic solvents.18b,^24^

## 3 Lewis Acid Catalysis in Aqueous or Organic Solvents

Although rate enhancement was found in the Mukaiyama aldol reaction in water, low yields and low reaction rates were often observed without activation by Lewis acids. On the other hand, traditional Lewis acids such as Al(III), Ti(IV), and Sn(IV) significantly accelerate the Mukaiyama aldol reaction in organic solvents, whereas these Lewis acids decompose rapidly in the presence of water. Indeed, Lewis acids were believed to be incompatible with water for a long time.

**Scheme 5:**
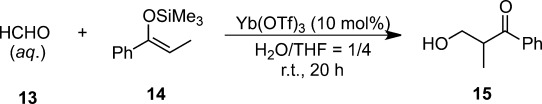
Yb(OTf)_3_-catalyzed Mukaiyama aldol reaction in aqueous media.

However, it was discovered in 1991 that lanthanide trifluoromethanesulfonates (triflates) [Ln(OTf)_3_] are water-compatible Lewis acids and accelerate the Mukaiyama aldol reaction significantly in aqueous media (water/organic solvents).^25^,^26^ For example, Yb(OTf)_3_, a representative lanthanide triflate, was revealed to exhibit superior performance in the Mukaiyama aldol reaction of various silicon enolates with aqueous formaldehyde **13** in water-tetrahydrofuran (THF) (Scheme 5).^27^ The reactions also proceeded well in water-ethanol-toluene cosolvent systems.^28^

**Scheme 6:**
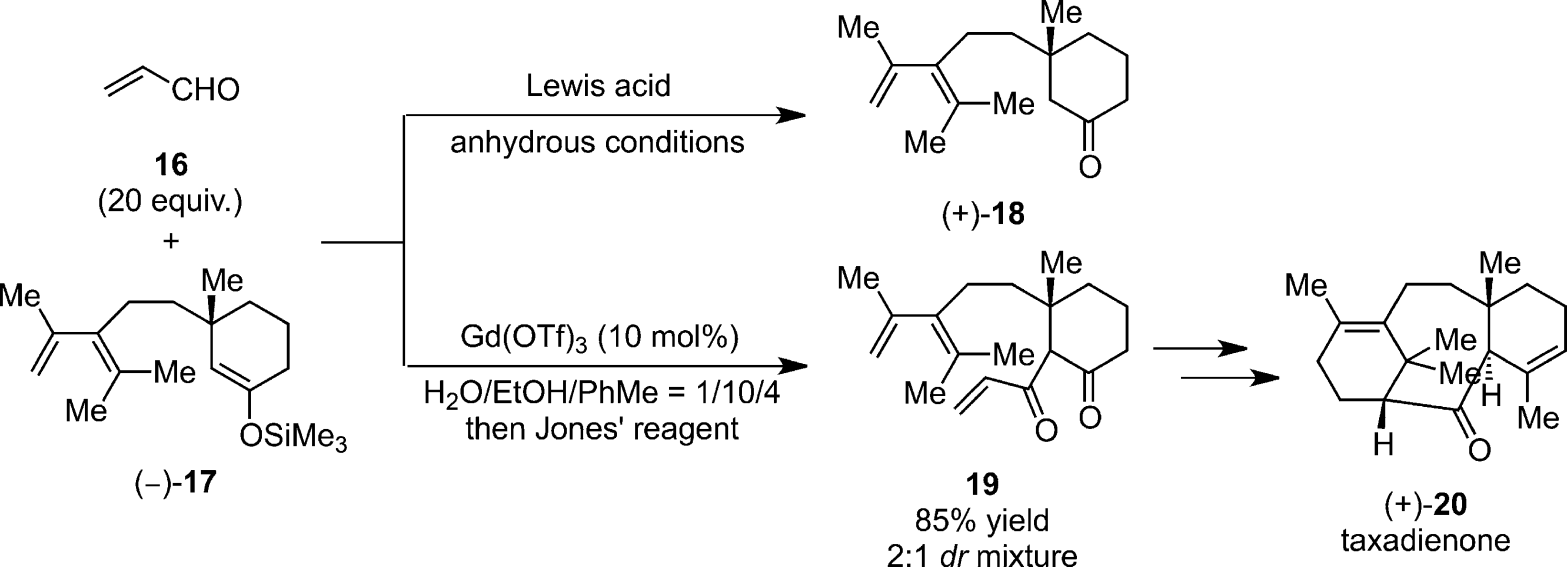
Mukaiyama aldol reaction of **17** with acrolein **16**.

A wide variety of aldehydes other than formaldehyde could also be activated successfully by lanthanide triflates to give cross-aldol adducts in high yields. Even water-soluble aldehydes such as acetaldehyde, acrolein, chloroacetaldehyde, and aldehydes possessing coordinating heteroatoms such as salicylaldehyde and 2-pyridinecarboxyaldehyde reacted successfully.^29^ The reactions carried out without water failed to give the corresponding aldol products, thus verifying the intriguing influence of water on these catalysts.^30^ Moreover, water-compatible Lewis acid catalysis may offer many advantages over traditional TiCl_4_-promoted reactions performed under strictly anhydrous conditions. For example, performing the Mukaiyama aldol reaction in aqueous media enabled the use of aqueous formaldehyde as a C_1_ source. In general, hydroxymethylation reactions using formaldehyde as one of the most valuable C_1_ electrophiles have received immense attention in organic synthesis.^31^ Although formaldehyde gas and paraformaldehyde in organic solvents can be employed as C_1_ electrophiles, tedious and harmful procedures to generate the formaldehyde monomer from oligomers^32^ are the main disadvantage. Ln(OTf)_3_-catalyzed hydroxymethylation has been employed to construct complex molecules in the total synthesis of natural compounds such as A-seco-taxane^33^ and (−)-sclerophytin A.^34^ Moreover, practical attachment of a hydroxymethyl function on the α-carbon adjacent to the carbonyl group has been enlisted in the total synthesis of biologically active compounds such as diazonamide A,^35^ (−)-strychnine,^36^ and acutifolone A,^37^ in which the initial formation of silicon enolates (TMSCl, Et_3_N) served to create latent nucleophiles that were subsequently unleashed upon Ln(OTf)_3_-catalyzed reactions with aqueous formaldehyde. Such a two-step, one-pot procedure was utilized in a δ-lactone synthesis protocol.^38^ Furthermore, this aqueous strategy impedes a seriously destructive desilylation pathway. The formation of an undesired desilylated ketone was often the sole pathway when attempting the Mukaiyama aldol reaction with a conventional methodology. For instance, the Mukaiyama aldolization of TMS enolate **17** with acrolein **16** towards a total synthesis of taxanes has resulted in the sole formation of **18** in the presence of any catalyst.^39^ Gratifyingly, the desired reaction proceeded smoothly with Gd(OTf)_3_ in a water-ethanol-toluene system (1:10:4) to afford the corresponding keto-enone **19** as a 2:1 mixture of diastereomers after subsequent oxidization with Jones’ reagent (Scheme 6). This result bears an eloquent testimony to the overwhelming ascendancy of the aqueous Ln(OTf)_3_ system as a powerful catalyst for the Mukaiyama aldol reaction.

**Scheme 7:**
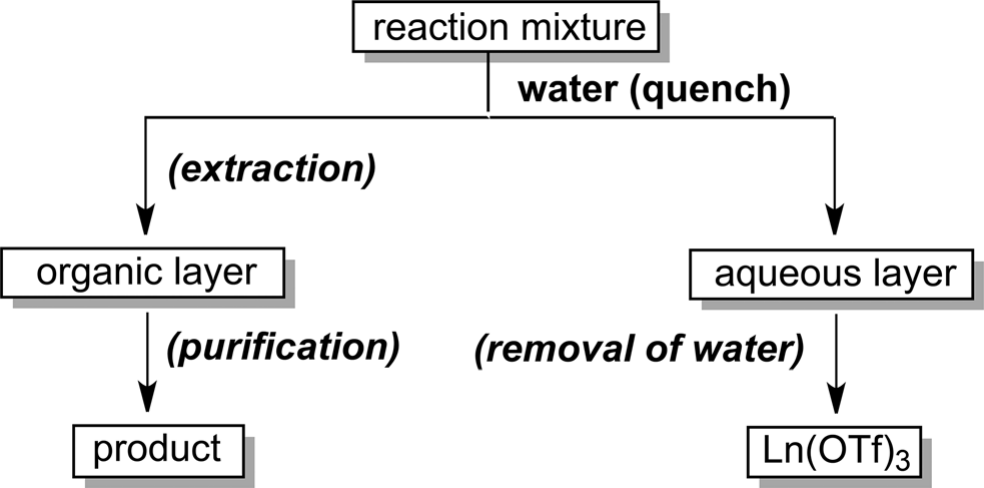
Recovery of the catalyst.

Another striking feature of Ln(OTf)_3_ is the ease of recovery from the reaction mixture. Because these metal triflates are soluble in water, they can be recovered quantitatively from the aqueous layer and the product can be obtained through simple extraction from the organic layer (Scheme 7).

### 3.1 Water-Compatible Lewis Acids

The discovery of rare earth triflates [Sc(OTf)_3_, Y(OTf)_3_, and Ln(OTf)_3_] as water-compatible Lewis acids was contrary to the conventional understanding that Lewis acids decomposed in the presence of water. A question was why Ln(OTf)_3_ is compatible with water, unlike conventional Lewis acids. Another question is whether there are any other water-compatible Lewis acids besides Ln(OTf)_3_. To answer these questions, extensive research has been conducted that has led to an expansion of the availability of water-compatible Lewis acids and catalysts. The reaction of benzaldehyde with propiophenone-derived silicon enolate **14** was chosen as a model and group 1–15 metal chlorides, perchlorates and triflates were screened in a water-THF cosolvent system.^40^ Selected results are shown in Table 2a. The reaction catalyzed by other metal salts hardly provided any of the desired product **21**.

**Table 2:** (a) Evaluation of catalysis by different Lewis acids in the Mukaiyama aldol reaction. (b) Hydrolysis constants and exchange rate constants for substitution of inner-sphere water ligands

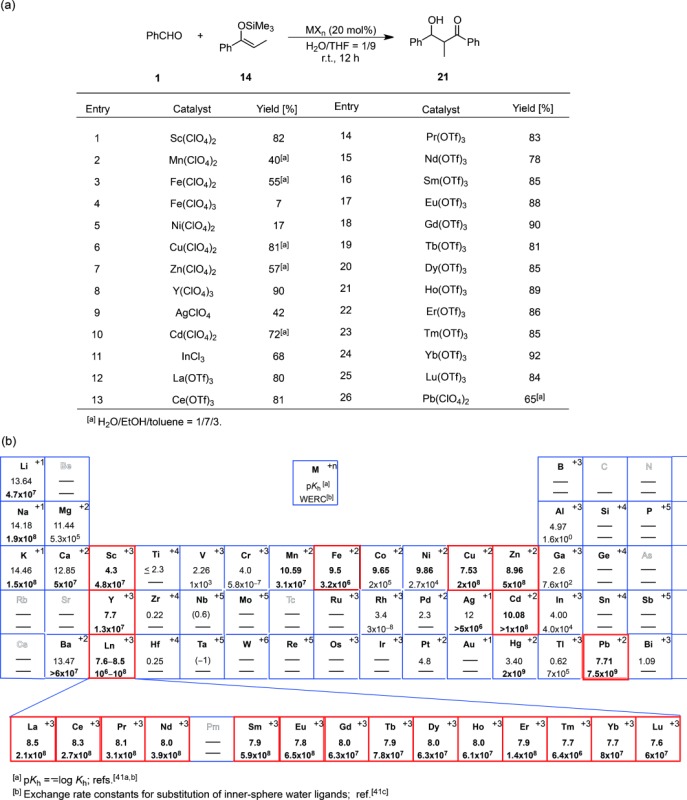

**Scheme 8:**
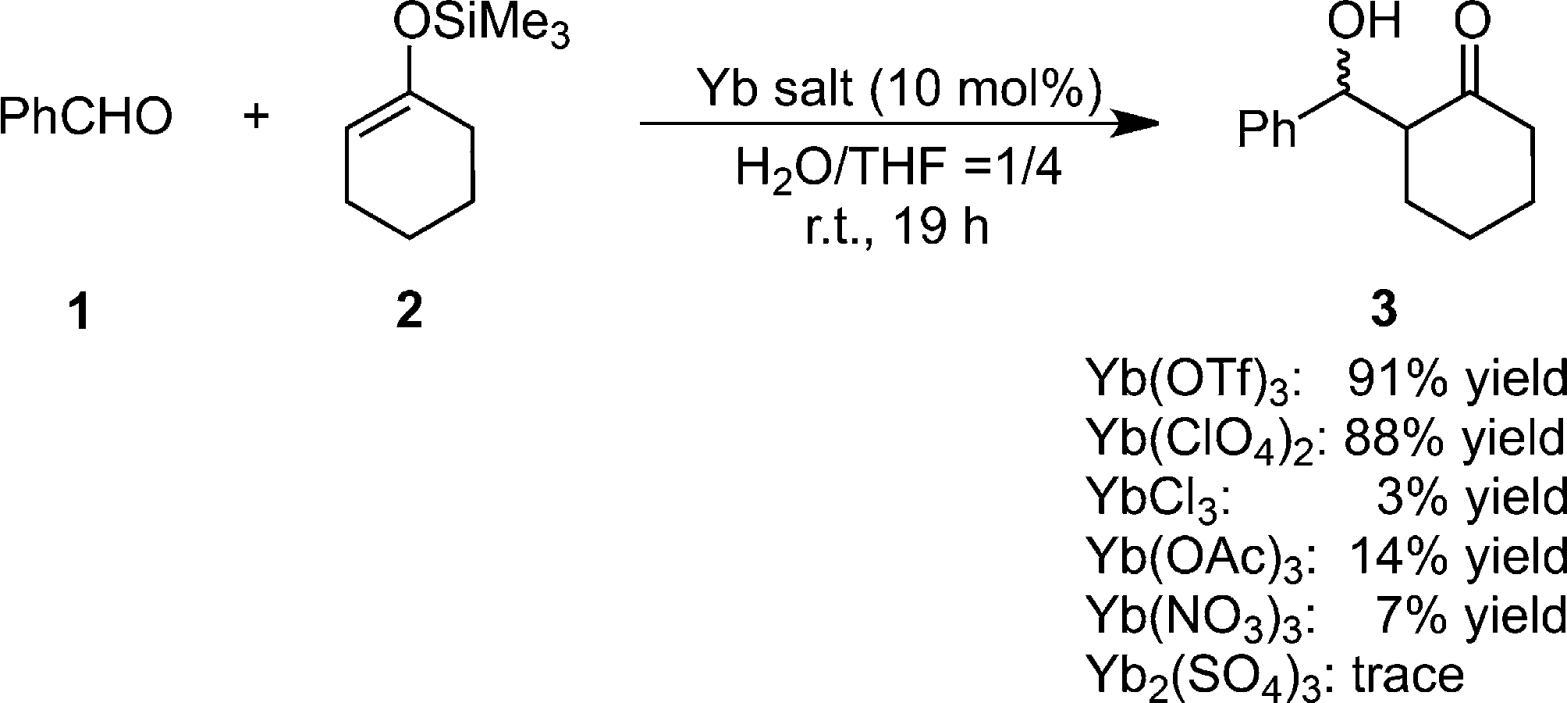
Effect of Yb salts.

In addition to rare-earth metal cations, Fe(II), Cu(II), Zn(II), Cd(II), and Pb(II) were found to function as efficient and promising Lewis acids in aqueous media, and this necessitated the establishment of the criteria for catalytic activity of the metal cations. Superior catalytic activity was observed in the presence of the metal cations surrounded by red squares in Table 2b. Given the correlation between these metal cations and catalytic activity, hydrolysis constants (*K*_h_) and exchange rate constants for the substitution of inner-sphere water ligands [water exchange rate constants (WERC)] are suitable factors for estimating the catalytic activity of Lewis acids.^41^ These active metal compounds were found to have p*K*_h_ values from about 4 [4.3 for Sc(III)) to 10 (10.08 for Cd(II)] and WERC values greater than 3.2×10^6^ M^−1^ s^−1^. Cations with large p*K*_h_ values do not generally undergo efficient hydrolysis. Cations with p*K*_h_ values less than 4 are readily hydrolyzed to produce protons in sufficient numbers to cause rapid decomposition of the silicon enolate. On the other hand, when the p*K*_h_ value is higher than 10, the Lewis acidities of the cations are too low to catalyze the aldol reaction. To act as an efficient catalyst in water, large WERC values may be necessary to allow sufficiently fast exchange between the water molecules coordinated to the metal and the aldehyde substrate. “Borderline” species such as Mn(II), Ag(I), and In(III), with p*K*_h_ and WERC values close to the criteria limits, provide the aldol adducts in moderate yields. Whereas the precise activity of Lewis acids in aqueous media cannot be quantitatively predicted by p*K*_h_ and WERC values, these values have been instrumental in the identification of promising metal compounds as water-compatible Lewis acid catalysts,^42^ and have also provided mechanistic insights into Lewis acid catalysis in aqueous media. By taking into account the high coordination numbers of these effective metals, the phenomenal rate acceleration by water can be attributed to the predominant ionic properties of the interaction between the Lewis acidic metals and the counter anions. Indeed, the catalysis of ytterbium salts with more nucleophilic counter anions such as Cl^−^, OAc^−^, NO_3_^−^ and SO_4_^−^ was far less than the effective catalysis of ytterbium salts with less-coordinating counter anions such as OTf^−^ (91% yield, *syn*:*anti*=73:27) or ClO_4_^−^ (88% yield, *syn*:*anti*=76:24)29b (Scheme 8).

The best yields were obtained with 10–20% water. Water seems to destabilize silicon enolates through hydrolysis of ytterbium salts.^43^ The amount of water governs the stereochemical outcome as well as the consequent catalytic turnover in water/THF solution. While the reaction of benzaldehyde with cyclohexanone-derived silicon enolates proceeded with *anti*-preference in the absence of water, the stereochemistry of the product underwent a change toward *syn* conformations as the amount of water increased; however, the selectivity changed no further (up to *syn*/*anti*=73/27) when more than 15 equivalents of water were added.29b

An unequivocal interrelationship between the coordination environment of lanthanide triflates and the steady-state reaction rate of the Mukaiyama aldol reaction was also proven through luminescence-decay measurements in combination with high-performance liquid chromatography analyses.^44^ These phenomena can be explained as follows. The predominant coordination of a THF molecule to Yb(OTf)_3_ stabilizes the cyclic six-membered transition state that led to the lower activity and *anti*-selectivity in the reaction when no water or a small amount was added.^45^ On the other hand, when the equivalents of water are gradually increased, the coordination of water induces the naked active ytterbium cation with its high water-exchange rate constant^46^ to activate aldehydes effectively and catalyze the reaction *via* an acyclic transition state.^47^ That is, the structural fluctuation of coordination water molecules around lanthanide ions determines favorable transition states (TS) in terms of entropy, promotes hydration of the silicon enolate and proton transfer to the aldehyde, and activates both substrates; this was confirmed by the AFIR method.^48^ Thus, the elaborate exploitation of the unique properties of water in irreversible Mukaiyama aldol reactions is expected to facilitate the catalytic turnover with simultaneous desilylation as a direct access to aldol adducts, while in conventional acid- or base-catalyzed aldol reactions, the reaction yields of the aldol adducts are destined to depend upon their thermodynamic stabilities because of the reversibility of the reactions.

**Scheme 9:**
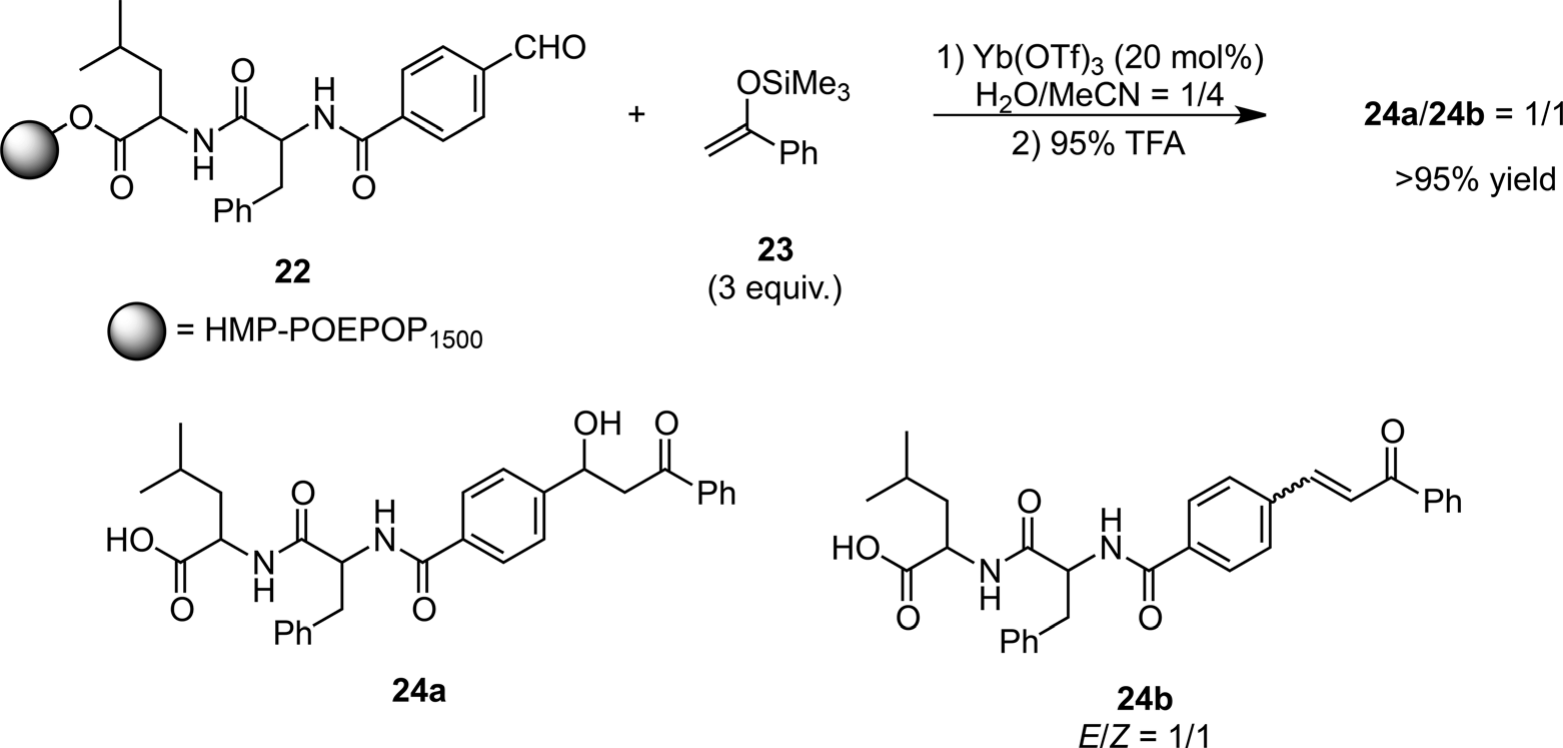
Solid-phase Mukaiyama aldol reactions of a model peptide substrate.

The emergence of water-compatible Lewis acids has definitely broken down the wall of traditional prejudice that water is a detrimental contaminant in organic synthesis, and has also offered industrially beneficial methodologies that facilitate recovery and reuse of these catalysts. As for heterogeneous catalysis, polar polyoxyethylene-polyoxypropylene (POE-POP) resin, derivatized with a 4-hydroxymethylphenoxy linker, was employed as a solid support for Ln(OTf)_3_-catalyzed Mukaiyama-type solid-phase aldol reactions.^49^ The utilization of an aqueous medium was crucial for the acquisition of sufficient reactivities and high yields were attained for an N-terminal peptide aldehyde substrate **22** (Scheme 9).

A series of fluoroalkyl end-capped 2-acrylamido-2-methylpropanesulfonic acid polymer [R_F_-(AMPS)_n_-R_F_; R_F_=fluorinated group such as CF(CF_3_)OC_3_F_7_] hydrogels containing Sc(OTf)_3_ was employed in the Mukaiyama aldol reaction in aqueous methanol.^50^ A gelation was derived from the synergistic interaction between the aggregation of end-capped fluoroalkyl segments and the ionic interactions of the betaine segments in water under non-cross-linked conditions.^51^ Not only did the reaction proceed smoothly, but the catalyst was also recovered quantitatively and the catalytic activity of the recovered catalyst was unaffected. Evaluation of homogeneous Lewis acid catalysis by water-soluble sodium salts or lithium salts of the sandwich-structured Hf/Zr-containing Keggin and Dawson POMs showed high *anti*-preference for Mukaiyama aldol reactions in an aqueous/CH_3_CN system.^52^

**Scheme 10:**
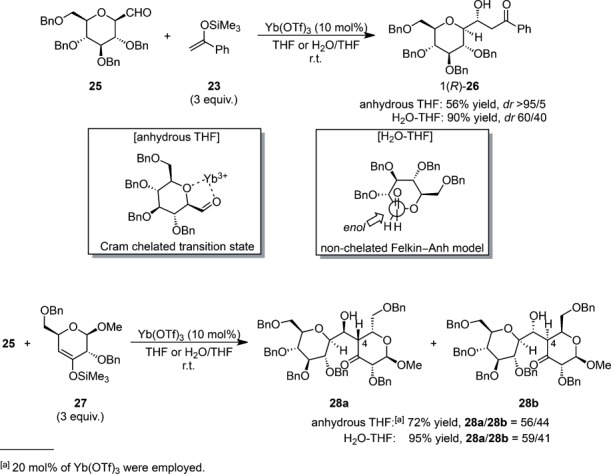
Synthesis of *C*-glycosides and *C*-disaccharides using Mukaiyama aldol reaction in aqueous media.

Lewis acid-catalyzed Mukaiyama aldol reactions in aqueous media could also be efficiently applied to the preparation of *C*-glycosides and *C*-disaccharides starting from formyl-2,3,4,6-tetra-*O*-benzyl-β-d-glucopyranoside **25** (Scheme 10).^53^ Yb(OTf)_3_ in aqueous media led to the aldol adducts in high yields with moderate diastereoselectivities for the synthesis of *C*-glycosides. Interestingly, a significant drop in reactivity as a single isomer was observed when the reaction was performed in anhydrous THF. The better diastereoselectivity observed in anhydrous THF could be rationalized from the Cram cyclic chelated model, in which the ytterbium atom may coordinate the carbonyl group and the endocyclic α-oxygen atom of the sugar moiety. In aqueous media, the attack of the electrophile may not be preferentially directed to one of the π-faces of the enolate, as depicted in the non-chelating Felkin–Anh model. In the Mukaiyama aldol reaction of the sterically hindered silicon enolate **27**, a low degree of diastereoselection was observed for the C-4 alkylation, which might be attributed to the poor steric control induced by the substituents adjacent to the reactive center (at the C-6 and C-2 positions).

Thermal treatment of montmorillonite K10 was also applicable to the Mukaiyama aldol reaction in water.^54^ The catalytic activity was attributed to the structural features of the clay and its inherent Brønsted acidity.

## 4 Lewis-Base Catalysis in Aqueous or Organic Solvents

**Scheme 11:**
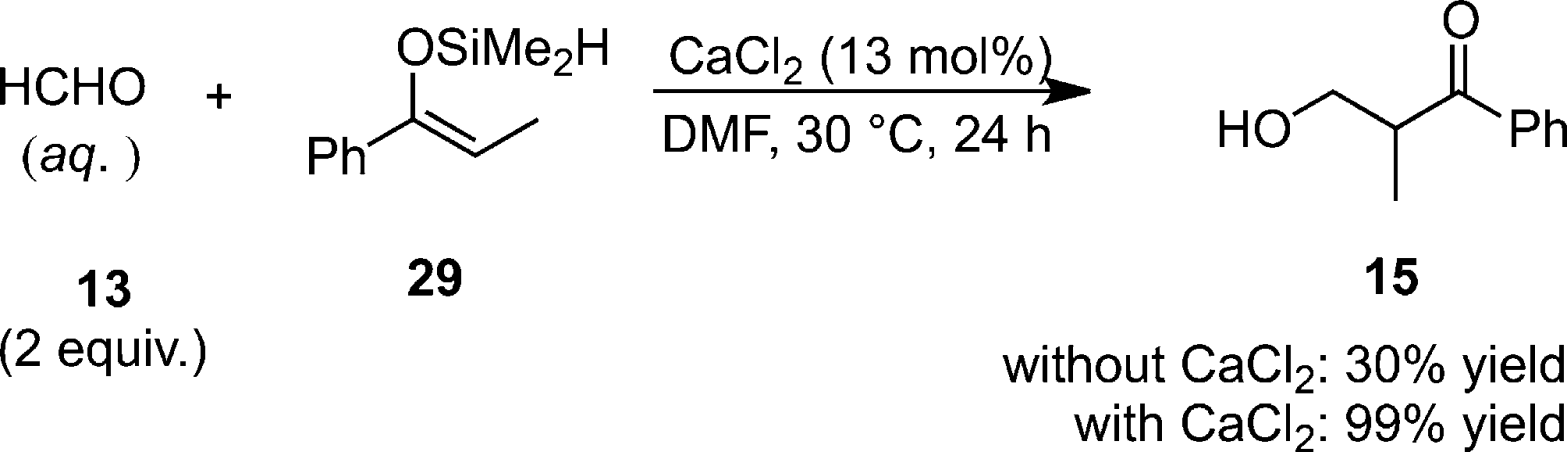
CaCl_2_-catalyzed Mukaiyama aldol reaction using aqueous formaldehyde.

Lewis-base catalysis can also apply to the Mukaiyama aldol reaction in the presence of water, albeit with a limited number of examples. It is known that trimethylsilyl enolates are not so strongly activated by non-ionic Lewis bases such as tertiary amines, *N*-oxides, or phosphine oxides.^55^ While one of the major problems was their low solubility in organic solvents such as THF and CH_3_CN, the use of protic solvents led to side reactions such as hydrolysis of the enolates.26a,^56^ Few of them were soluble in THF, toluene or CH_3_CN, but they provided considerable amounts of elimination products because of their strong Brønsted basicity.^57^ Hosomi et al. disclosed that the uncatalyzed reaction of benzaldehyde with dimethylsilyl (DMS) enolates proceeded under thermal conditions in DMF, while trimethylsilyl enolates hardly reacted.^58^ Extensive examination of additives to promote the Mukaiyama aldol reactions led to the discovery of calcium chloride as a catalyst, which effectively suppressed associated side reactions. The rate-accelerating ability of the calcium salt depends on the intrinsic nucleophilicity of the counter anion: TfO^−^<I^−^<Br^−^<Cl^−^. Tetrabutylammonium chloride also exhibited good catalytic activity for the Mukaiyama aldol reaction of DMS enolates. Given the diametrically different observations on rate acceleration between TMS and DMS enolates and the chloride ion’s higher nucleophilicity compared with those of bromide or iodide ions in an aprotic solvent such as DMF, and the low Lewis acidity of CaCl_2_,^59^ chloride ion was determined to function as a Lewis base to activate the DMS enolate. In spite of a much lower tolerance to water, the DMS enolate turned out to be a competent nucleophile in the presence of CaCl_2_ in water or aqueous DMF. Consequently, the CaCl_2_-promoted system could be utilized for the reaction of aqueous aldehydes such as aqueous formaldehyde, phenylglyoxal and chloral, and this was the first example of Lewis base-catalyzed Mukaiyama aldol reactions in aqueous media (Scheme 11).

**Scheme 12:**
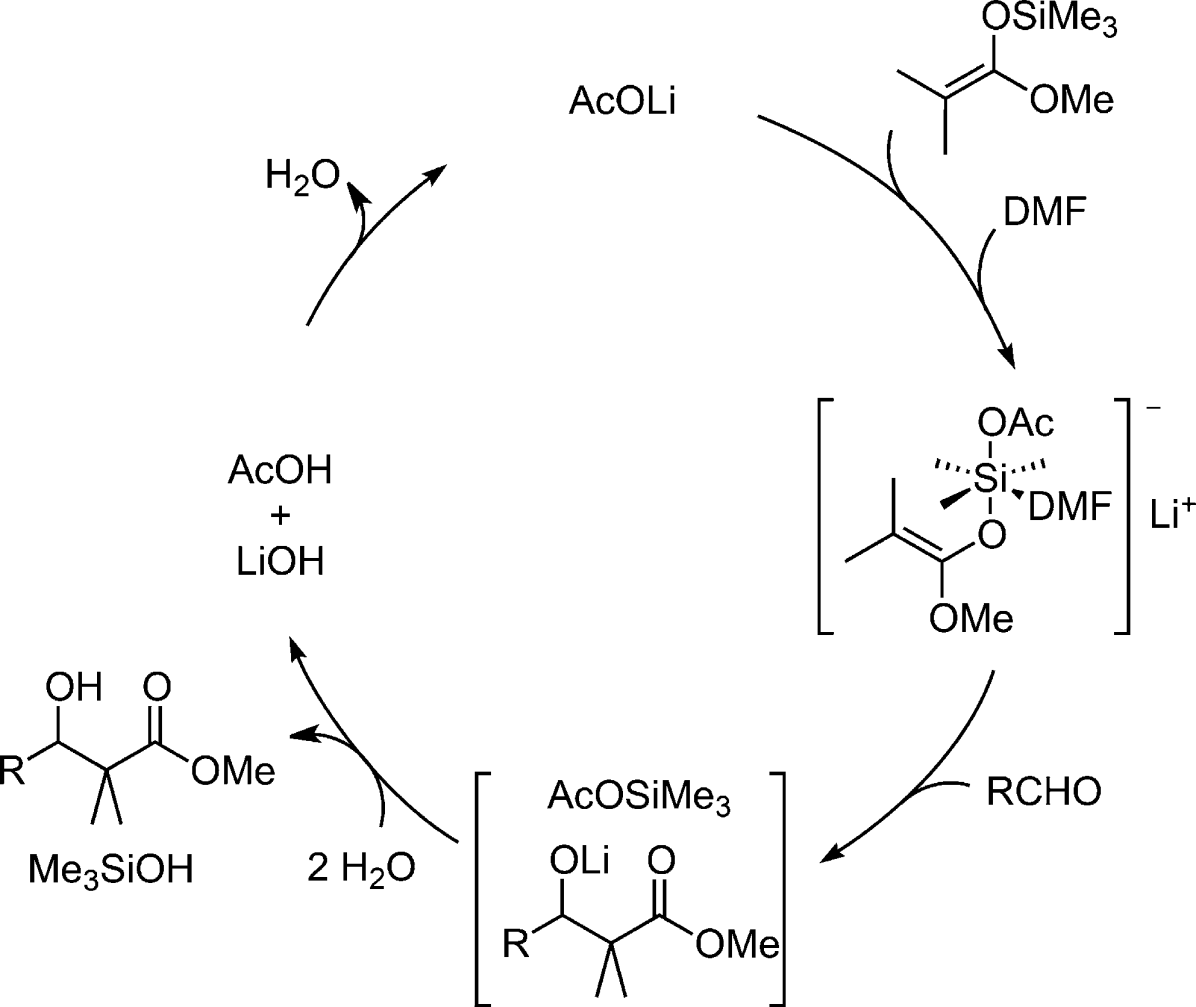
AcOLi-catalyzed mechanism for Mukaiyama aldol reaction in aqueous DMF.

Following their previous studies of the aldol reactions carried out under anhydrous conditions,^60^ Mukaiyama et al. reported that 10 mol% of lithium acetate could function as a Lewis base catalyst to catalyze the reaction between benzaldehyde and ketene silyl acetal at −45 °C in DMF:H_2_O=50:1 (Table 3).^61^ Several metal carboxylates, such as sodium acetate, lithium benzoate, and lithium acetate were found to act as catalysts. However, electron-poor benzoate suffered a significant drop in reactivity (entry 5). They assumed that the role of the carboxylate in the catalytic cycle was the formation of a lithium aldolate, *via* a hexacoordinated hypervalent silicate, that underwent rapid hydrolysis and subsequent neutralization to regenerate the lithium acetate catalyst (Scheme 12). The significant dependence of reactivity on the electrophilicity of the starting aldehydes supported their hypothesis.

**Table 3:** Lewis base-catalyzed Mukaiyama aldol reaction in aqueous DMF

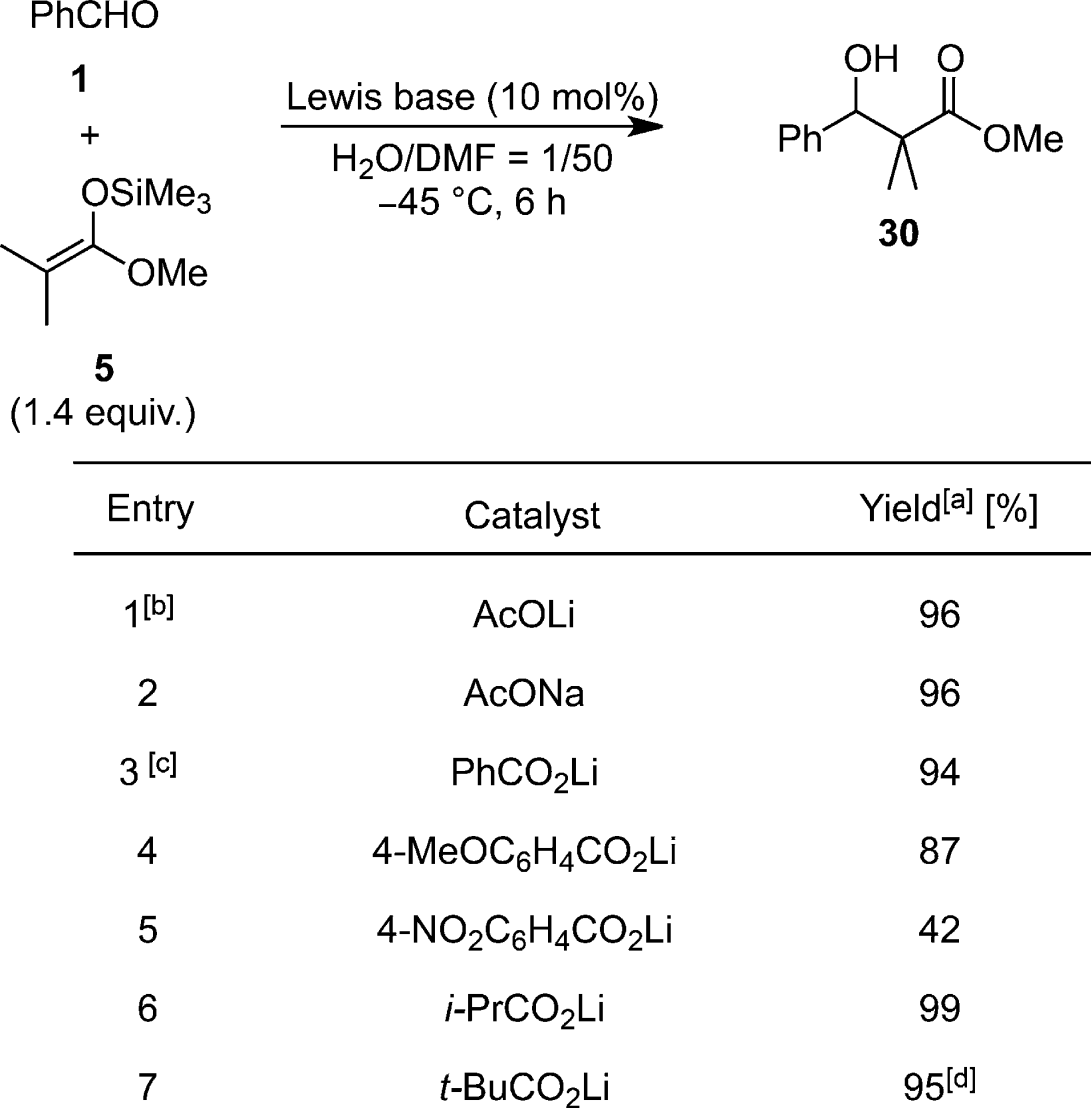

^[a]^ Determined by ^1^H NMR analysis.

^[b]^ 2 equiv. of **5** were added.

^[c]^ For 6.5 h.

^[d]^ Isolated yield.

Scandium fluoride (ScF_3_) was found to be a novel catalyst for the hydroxymethylation reaction of DMS enolates with aqueous formaldehyde in aqueous THF solution.^62^ Comparison of the reactivity between TMS and DMS enolates derived from propiophenone for hydroxymethylation reactions was examined using typical Lewis acids or bases (Table 4). The hydroxymethylation was sluggish without a catalyst (entry 1), whereas both enolates reacted to afford the product in the presence of a Lewis acid catalyst (scandium chloride); the TMS enolate providing a higher yield (entry 2). The products were also obtained from both enolates in the presence of KF and 18-crown-6 with the TMS enolate demonstrating slightly better reactivity (entry 3). The addition of HF resulted in lower yields with both enolates (entry 4). Interestingly, ScF_3_ produced the product only from DMS enolate (entry 5). This unique character of ScF_3_ denoted that its reactivity toward TMS and DMS enolates was different from that of conventional Lewis acid or fluoride ion^63^ (Lewis base)-catalyzed reactions. Some reports have shown that fluoride ions can activate silicon enolates effectively even in a protic solvent.^64^ The detailed mechanism of the preferential activation of DMS enolates by ScF_3_ remains unknown.

**Table 4:** Comparison of the reactivity of TMS or DMS enolates with various catalysts

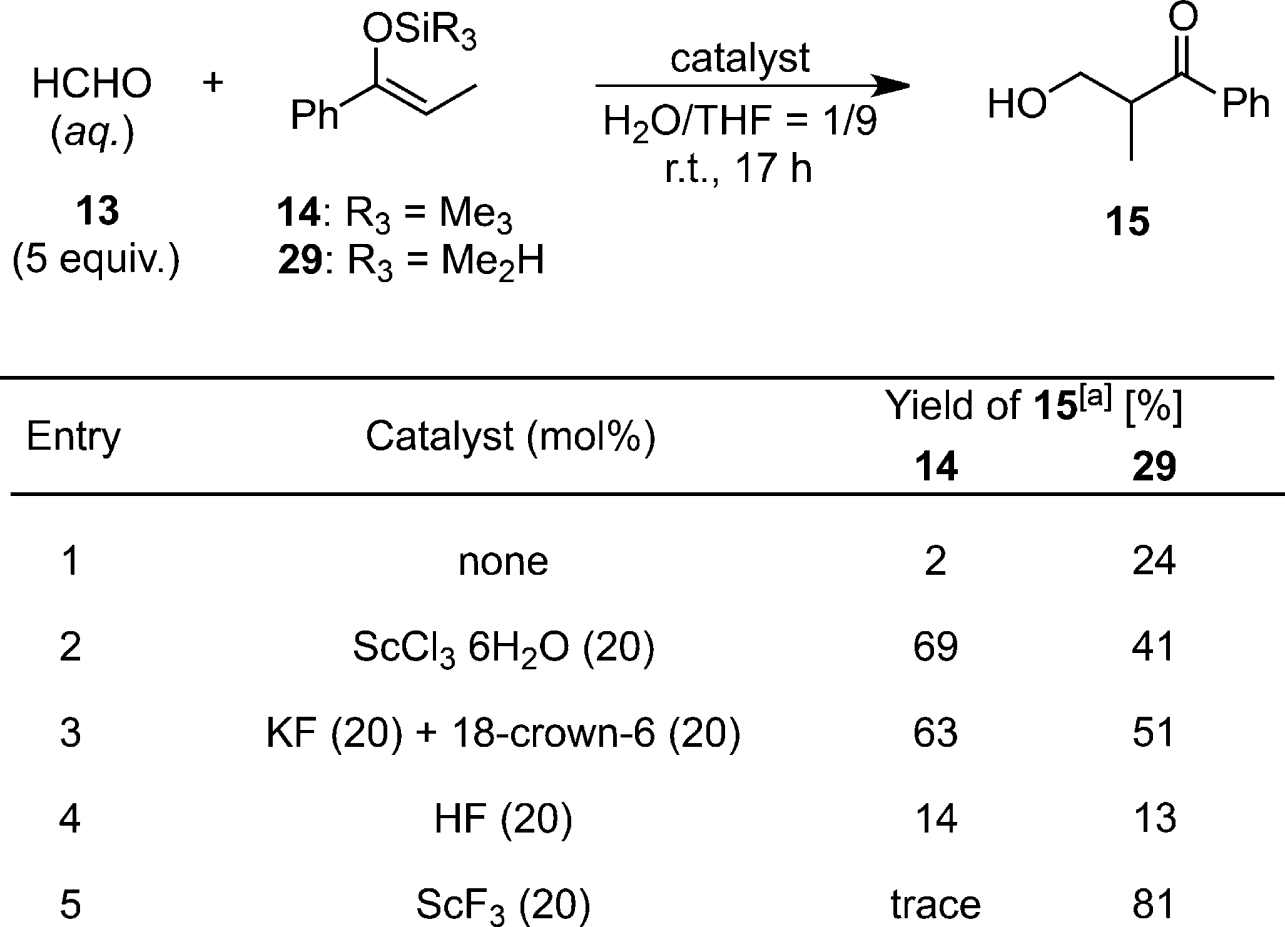

^[a]^ Isolated yield.

## 5 The Mukaiyama Aldol Reactions in 100% Water

A fundamental challenge of performing the Mukaiyama aldol reactions in water is the competition by rapid hydrolysis of silicon enolates, which can lower reactivity and narrow the substrate generality. To make the desired catalytic pathway dominant over the competitive hydrolysis, a possible approach is to enhance the hydrophobicity of the reaction environment. The addition of a catalytic amount of surfactant was found to increase the yield in Sc(OTf)_3_-catalyzed Mukaiyama aldol reactions in 100% water. Micellar systems containing anionic^65^ or non-ionic surfactant^66^ have led to remarkable enhancements of reactivity, even with ketene silyl acetals (Table 5).^67^ Sodium dodecyl sulfate (SDS) was the most effective. Only cetyltrimethylammonium bromide (CTAB) was found not to be an effective surfactant because of the possibility of hydrolysis of the silicon enolate.

**Table 5:** Effect of surfactants on Mukaiyama aldol reaction in water

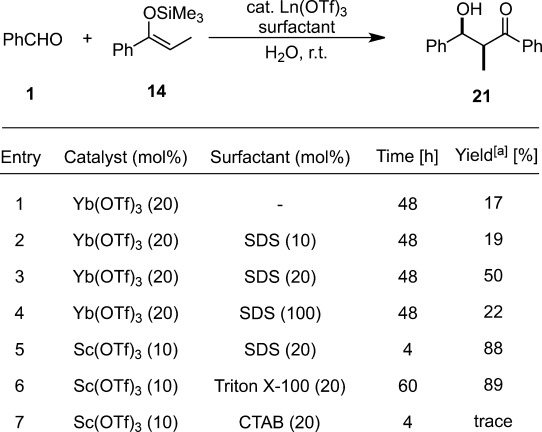

^[a]^ Isolated yield.

**Scheme 13:**
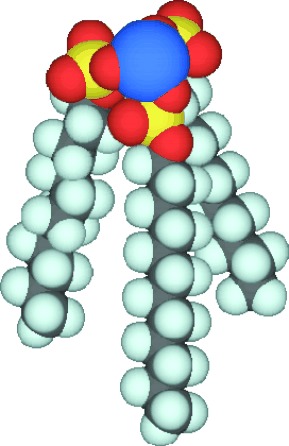
Schematic representation of scandium tris(dodecyl sulfate) (STDS).

A major disadvantage of Lewis acid catalysis aided by surfactants was that semi-catalytic use of the surfactants was required to achieve good results. There were also problems with separation. Because of the high affinity of water-compatible Lewis acids with water, the relative concentration of the catalyst in the hydrophobic micelles is low. Hence, there were severe substrate limitations in the Lewis acid-SDS system; very labile silicon enolates such as cyclohexanone-derived silyl enol ether decomposed rapidly. In 1998, a Lewis acid-surfactant combined catalyst (LASC) emerged as an innovative catalyst, in which a Lewis acid possessed ligands with surfactant properties to construct an efficient hydrophobic environment surrounding a Lewis acidic cation (Scheme 13).^68^,^69^

**Scheme 14:**
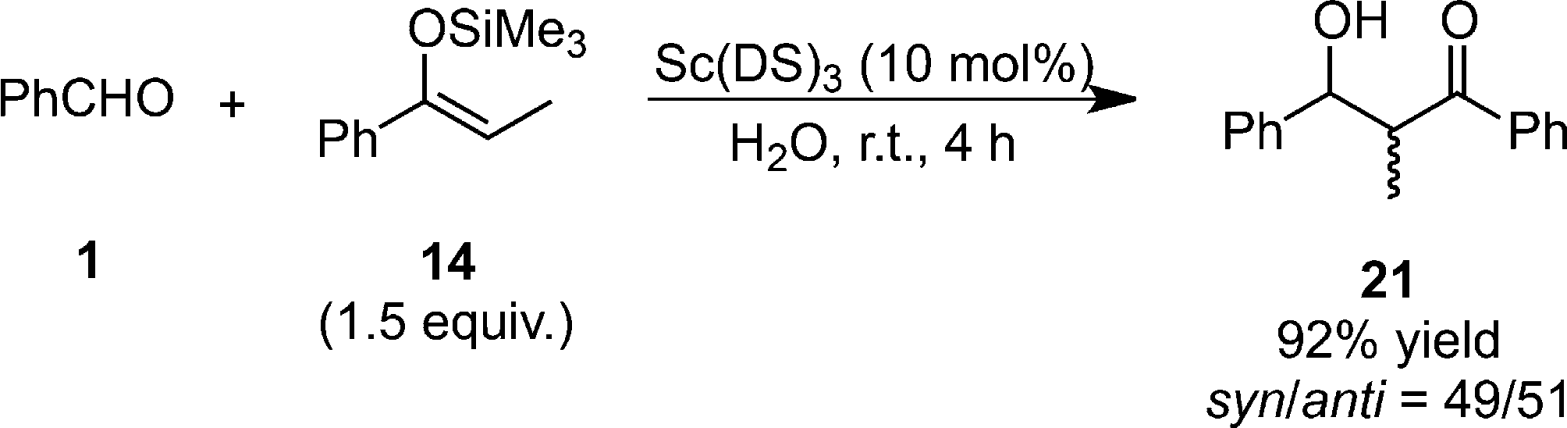
LASC-catalyzed Mukaiyama aldol reaction.

Scandium tris(dodecyl sulfate) [STDS, Sc(DS)_3_ (DS=OSO_3_C_12_H_25_)] was shown to dissolve in water and after the addition of organic substrates, a white dispersion formed. These oily particles, consisting of the organic substrates and STDS, were stabilized and dispersed in water to promote Mukaiyama aldol reactions (Scheme 14). Because Sc(OTf)_3_ *does not* react with SDS to form STDS *in situ*, the catalytic efficiency of STDS was superior to that of the Sc(OTf)_3_-SDS system.69a Indeed, the Sc(OTf)_3_-SDS system makes a kind of micelle (not a dispersion).

The use of STDS to construct highly reactive microenvironments in water enlarged the substrate scope in the Mukaiyama aldol reaction to include quite labile silicon enolates. Indeed, the Mukaiyama aldol reaction was found to proceed 5×10^3^ times faster in water than in dichloromethane.69a It was also shown that multifarious LASCs were applicable to various reactions in water.^70^,^71^ In aqueous solution, surfactants aggregate into micelles, where the hydrophobic portions of the molecules are protected from water.^72^ Normally, micelles in aqueous solution form with the surfactant molecules orienting themselves into spherical or elliptical structures with their hydrophobic tails oriented toward the center and their hydrophilic heads oriented toward the surrounding water.

**Scheme 15:**
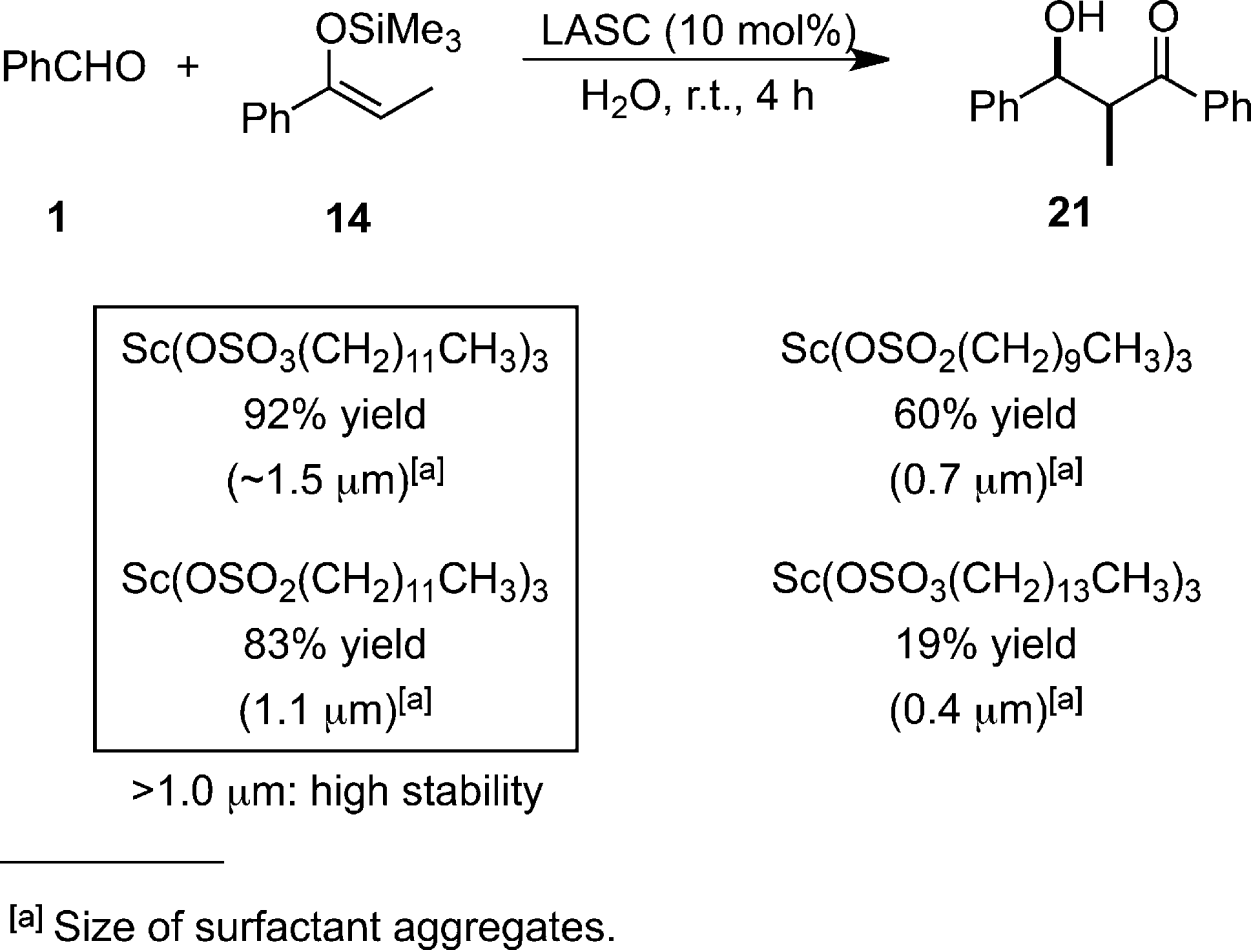
Correlation between size of colloidal dispersion and reactivity.

Elaborate regulation of reaction environments in micellar catalysis has been elucidated through many mechanistic insights. An NMR study disclosed that the micelles formed by SDS had approximately one-third of their surface covered by the hydrophilic head groups while the remaining two-thirds were covered by the hydrocarbon tails.^73^ The formation of micelles can be explained by thermodynamics: they can form spontaneously as a result of a balance between entropy and enthalpy. Despite the reduction in entropy caused by surfactant aggregation in water, the hydrophobic effect is a driving force for micelle formation. Micelles do not form unless the concentration of a surfactant is greater than the critical micelle concentration (CMC) and there exists a critical temperature (Krafft temperature) above which the solubility rapidly increases (to become equal to CMC).^74^ Theoretically, the CMC decreases as the polar head becomes smaller and as the alkyl chain length increases. Indeed, an NMR study of SDS micelles labeled with paramagnetic ions such as Co(DS)_2_ revealed that the charged surface was smooth and the chains minimized their radial extension.^75^ The correlation between the size of colloidal dispersions measured by dynamic light scattering (DLS) and their reactivity was evaluated for Sc-based LASCs (Scheme 15).^76^ When the catalyst possesses a lower CMC, for instance Sc(OSO_2_C_14_H_29_)_3_, the Mukaiyama aldol reaction proceeded less smoothly. Because of aggregation of hydrophobic substrates, the dispersion system was stable for only a few minutes and quick aggregation took place. Because typical emulsion sizes created in water are 1 μm, this low stability of the dispersion might be the reason for the low yields observed. All the particles formed from the mixture of Sc(OSO_2_C_12_H_25_)_3_ and benzaldehyde in water have a diameter of 1 μm and the molecular area of Sc(OSO_2_C_12_H_25_)_3_ was determined to be 132 Å^2^. Based on these facts, only approximately 0.08 mol% Sc(OSO_2_C_12_H_25_)_3_ with respect to benzaldehyde is sufficient to form monolayers around the aldehyde. This means that excess LASCs should be stacked at the interface between the water and the benzaldehyde phase in the presence of more than 0.08 mol% Sc(OSO_2_C_12_H_25_)_3_. As mentioned above, the remarkable enhancement of reactivity by Brønsted acids was observed in LASC-mediated Mukaiyama aldol reactions in water (see Table 6).^77^

**Table 6:** Effect of Brønsted acids on asymmetric Mukaiyama aldol reactions in water

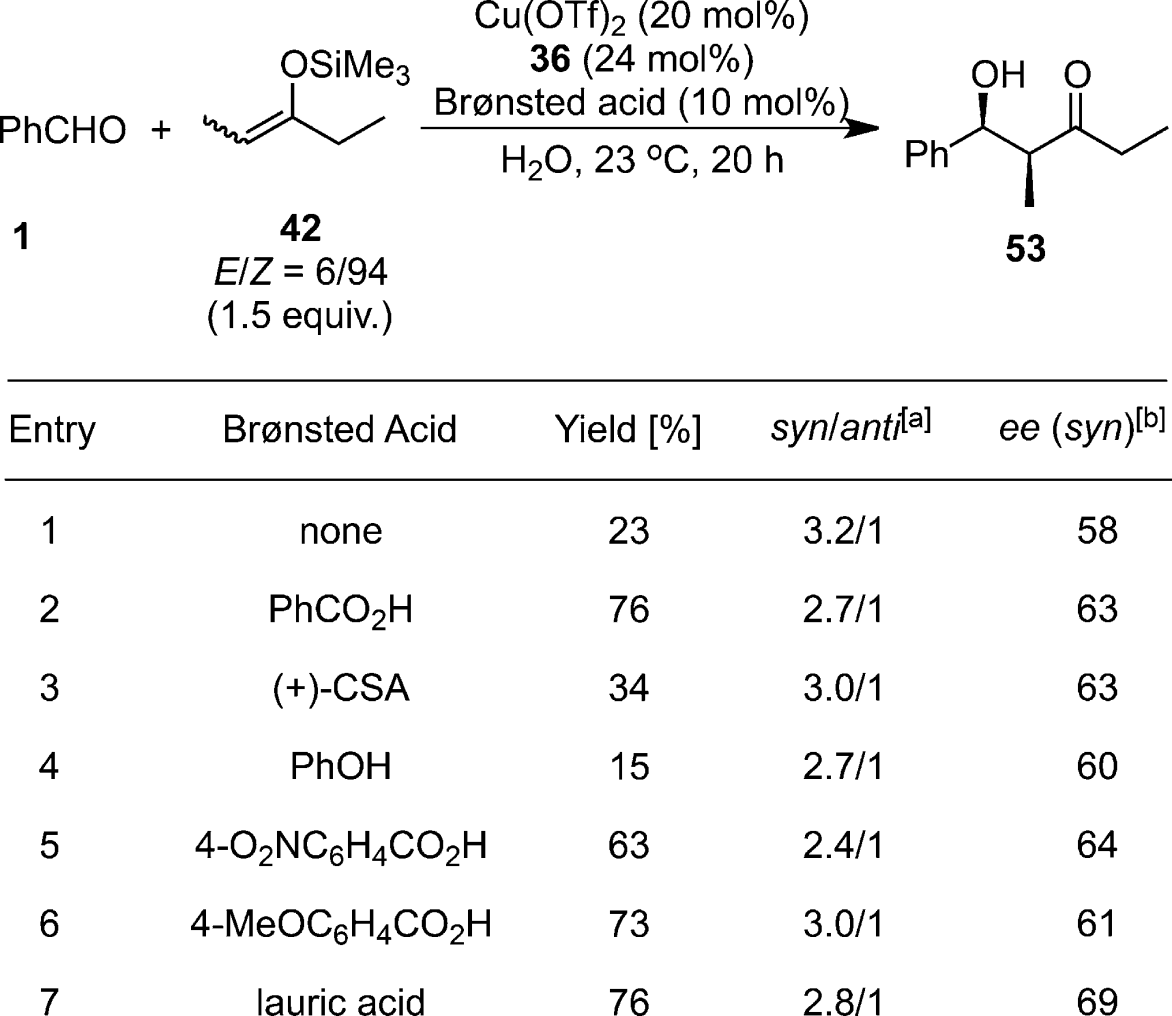

^[a]^ Determined by NMR analysis.

^[b]^ Determined by chiral HPLC analysis.

**Scheme 16:**
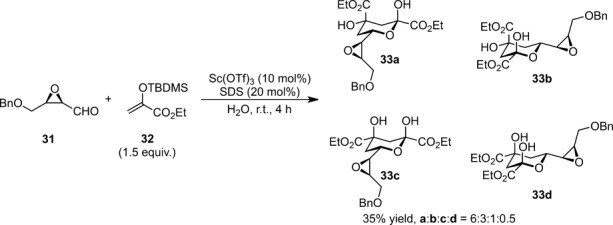
Aqueous Mukaiyama aldol reaction of α,β-epoxy aldehydes.

The reactions of *cis* and *trans* α,β-epoxy aldehydes with ketene silyl acetals proceeded using the Sc(OTf)_3_-SDS system in water were studied.^78^ The use of cosolvents failed to generate the aldol adducts, while the best results were obtained when operating under the micellar system with high *anti*-diastereofacial preference. The reaction of benzylated epoxy aldehyde **31** with ketene silyl acetal **32** derived from ethyl pyruvate led to ulosonic ester derivatives **33** (Scheme 16).

Polymer-supported (PS) catalysts were developed for the Mukaiyama aldol reactions in aqueous environments. Kobayashi′s group modified their previously reported LASC system by introducing polymer chain spacers as the hydrophobic component to the metallosurfactant catalyst.^79^ The reaction of benzaldehyde with 1-ethylthio-1-trimethylsiloxy-2-methyl-1-propene proceeded almost quantitatively in the presence of 3.2 mol% of the heterogeneous catalyst, and wider substrate generality was achieved by further modification.^80^ The catalytic activity of PS-Sc was dependent upon the hydrophobicity in water and was thus superior to that of STDS.

A periodic mesoporous Lewis acid catalyst [(OTf)_2_Sc-OSO_2_C_6_H_4_-PMO] exhibited catalytic activity and selectivity in aqueous Mukaiyama aldol reactions and compared favorably with a homogeneous catalyst Sc(OTf)_3_ or mesoporous SBA-15^81^-supported scandium triflate catalyst [(OTf)_2_Sc-OSO_2_C_6_H_4_-SBA-15].^82^ Hydrophobicity tests and substrate adsorption experiments demonstrated that its unique catalytic performance was related to the combined advantage of mesoporosity and a hydrophobic microenvironment, which effectively stabilized and concentrated the substances as well as decreasing the intrinsic mass-transfer resistance. The periodically arranged Lewis acids in the mesoporous silica framework inhibited leaching of metals from the active sites, leading to its high catalytic recyclability in water.

**Scheme 17:**
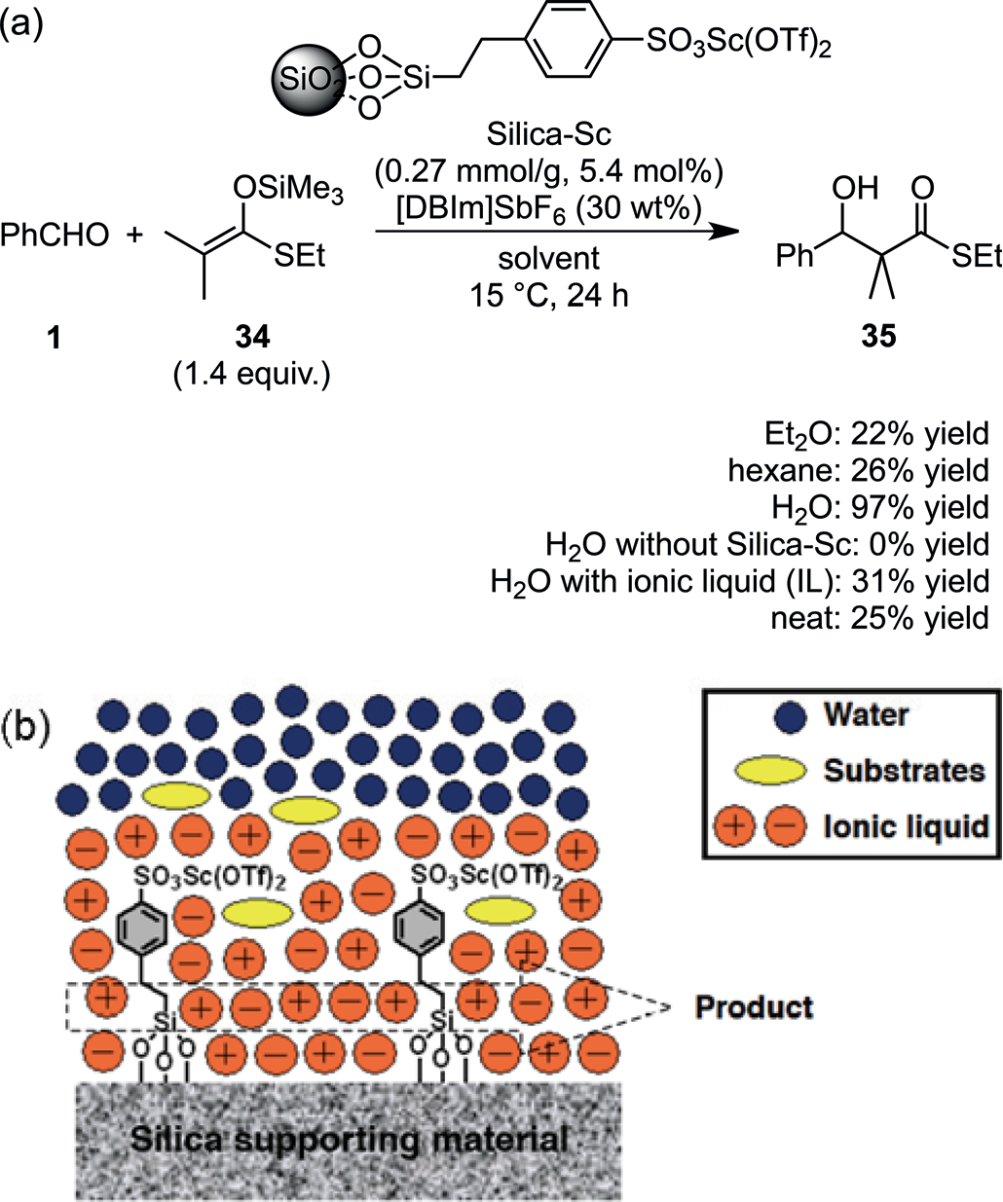
(a) Silica-supported scandium with ionic liquid (Silica-Sc-IL). (b) Hydrophobic reaction environment constructed by Silica-Sc-IL.

A heterogeneous scandium catalyst coated with an ionic liquid, [DBIm]SbF_6_, Silica-Sc-IL, also worked efficiently in the Mukaiyama aldol reaction (Scheme 17a).^83^ The reaction rate was much faster in water than in organic solvents or under neat conditions. It was determined that ionic liquids played a crucial role in the construction of efficient hydrophobic environments in water (Scheme 17b). Asymmetric catalysis could be also realized with this heterogeneous catalyst (*vide infra*).

**Scheme 18:**
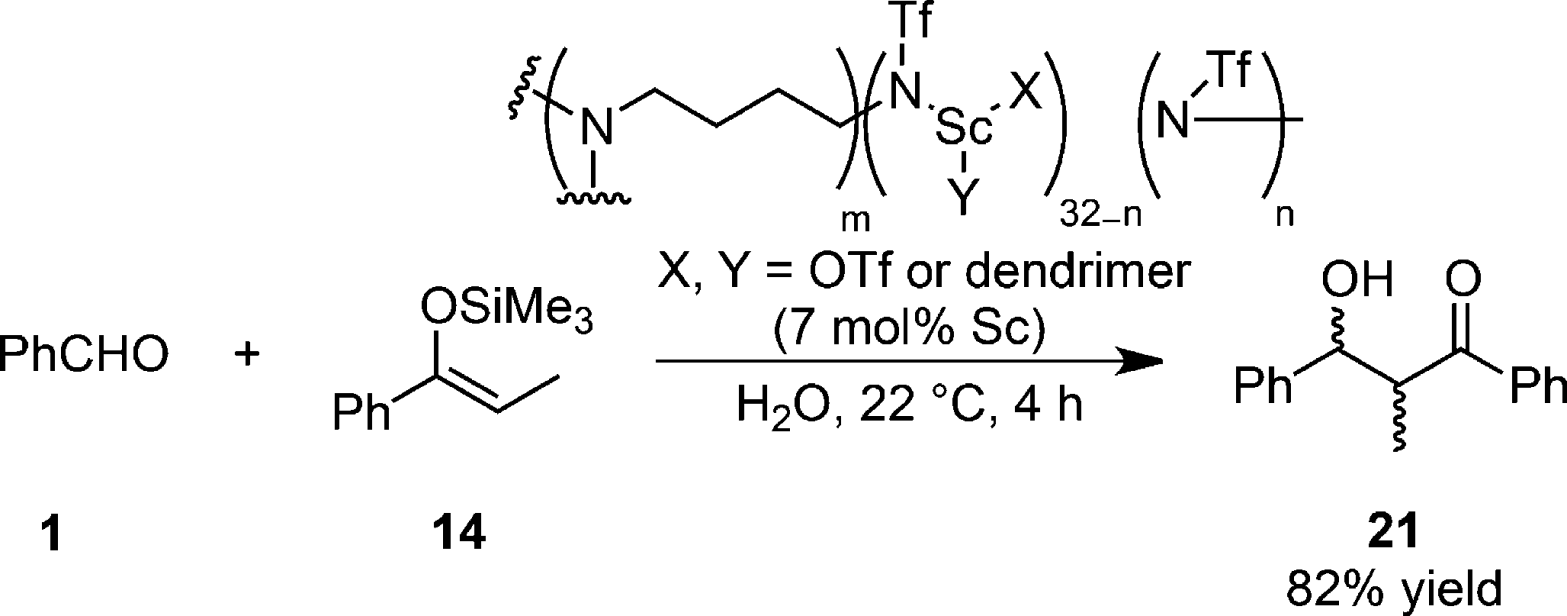
Cross-linked scandium-containing dendrimers.

Cross-linked, scandium-containing dendrimers derived from poly(amino) dendrimer as heterogeneous catalysts with Lewis acidic properties were reported to catalyze Mukaiyama aldol reactions in water (Scheme 18).^84^ Despite the heterogeneous material being non-porous, catalysis was achieved and the catalytically active scandium centers were accessible for the reaction to occur. A nanostructured, polymer-supported Sc catalyst utilizing a cross-linked inverted hexagonal lyotropic liquid-crystal phase was prepared and utilized as an efficient catalyst for the aldol-type reaction.^85^ While Sc(OTf)_3_ or STDS exhibited no diastereoselectivity, the nanostructured scandium catalyst exhibited moderate *syn* preference (*syn*:*anti*=76:24).

As another measure for overcoming the competitive hydrolysis, calix[6]arene derivatives bearing sulfonate groups on the upper rim and alkyl groups on the lower rim were developed.^86^ These macrocycles stabilized labile silicon enolates to promote the Mukaiyama aldol reactions in aqueous media with slight *anti* selectivities (*syn*:*anti*=31:69 to 40:60). These structures could be regarded as bundles of aromatic anionic surfactants. Indeed, the combination of aromatic surfactants and aryl aldehydes was favorable for the activation in the hydrophobic cavities.^87^ The recyclable inclusion complex formed from cyclodextrin (CD) and ytterbium tris(perfluoroalkanesulfonyl)amide was also applied to the Mukaiyama aldol reaction with ketene silyl acetals in water, albeit with slightly lower reactivity than that in DCM.^88^ It was reported that because of hydrophobic interactions, CD formed inclusion complexes with the fluorocarbon surfactants.^89^ Because the chain length of the perfluorobutane group was nearly equal to the length of cyclodextrin ring, it was assumed that the metal was located just outside the hydrophobic pockets of CD.

On the other hand, FeCl_3_ was reported as a homogeneous catalyst for the Mukaiyama aldol reactions and the diastereoselectivities were found to be controlled kinetically in water in the presence of a surfactant.^90^ Better rate acceleration and higher diastereoselectivity were attained in water than in water-THF solutions.

**Scheme 19:**
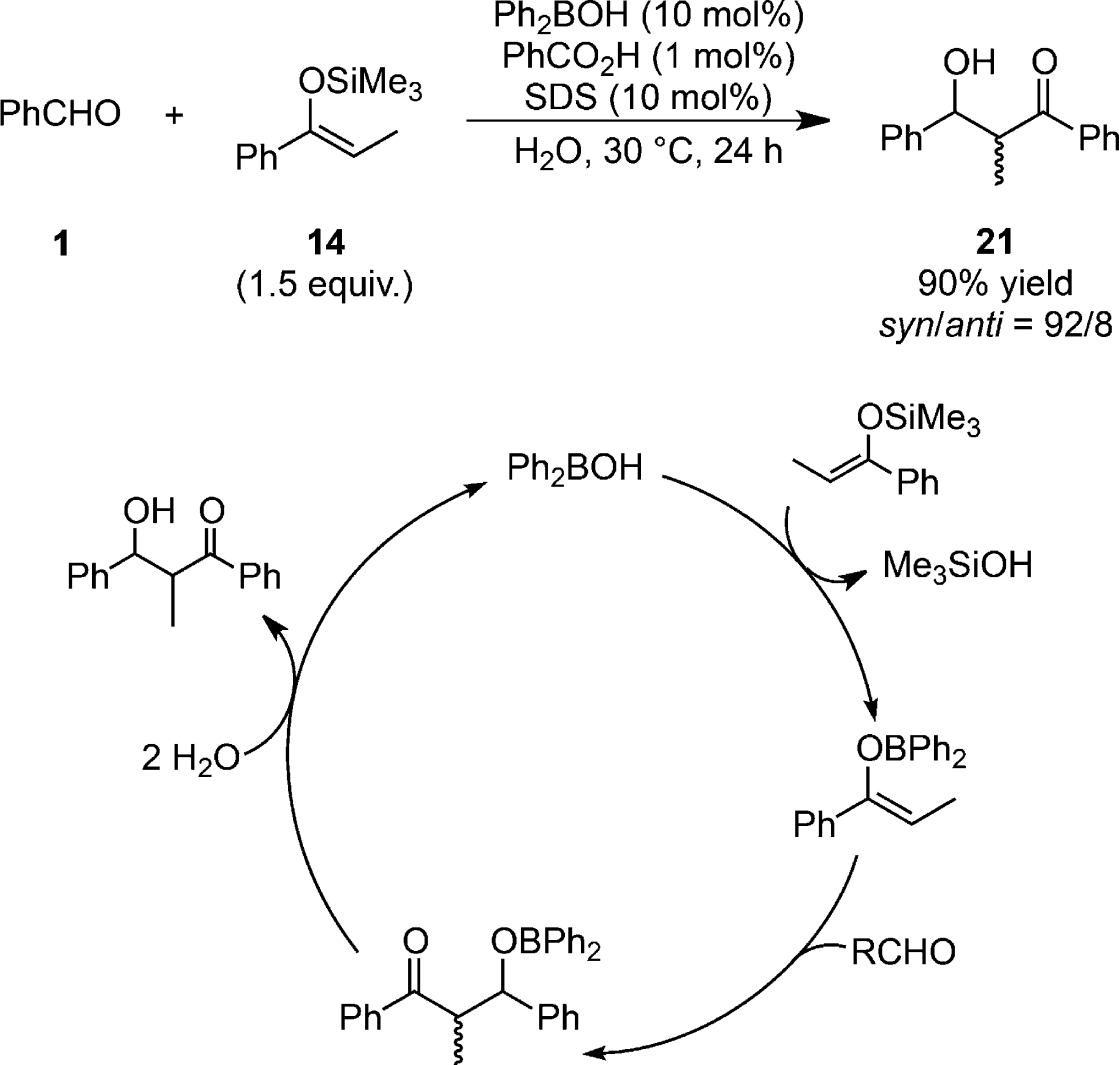
Diastereoselective Mukaiyama aldol reaction using diphenylboronic acid in water.

Diastereoselective aldol reactions have been successfully performed in water in the presence of a catalytic amount of a boron source (diphenylboronic acid).^91^ Only a trace amount of the product was obtained in organic solvents and a much lower yield than that in water was obtained under neat conditions (24% yield, *syn*:*anti*=90:10). It was found that the catalytic use of benzoic acid was greatly beneficial for this reaction (Scheme 19). When stronger acids such as hydrochloric acid or *p*-toluenesulfonic acid were employed, the yield decreased because of competitive hydrolysis of the silicon enolate and the diastereoselectivity was also lowered, presumably because of a Brønsted acid-catalyzed reaction pathway. A careful tuning of the diarylboronic acid structure led to the discovery that introducing a trifluoromethyl group at the *para* position brought about a significant rate acceleration compared with the unsubstituted molecule. Introduction of an electron-donating group at the *para* position reduced both the yield and selectivity, whereas high selectivity was maintained with electron-withdrawing substituents. The first-order kinetics with respect to the amount of silicon enolate supported the boron-enolate mechanism rather than the Lewis-acid mechanism (Scheme 15). The exchange from silicon to boron was assumed to be the rate-determining step. A significant dependence of the diastereoselectivity on the enolate geometry implied the formation of a chair-like six-membered transition state in the aldol condensation step. Although traditional boron-mediated aldol reactions required lower temperatures and strictly anhydrous conditions, this methodology enabled the aldol reaction to be performed in water at ambient temperature.

## 6 Asymmetric Catalysts in Aqueous Media and Water

**Scheme 20:**
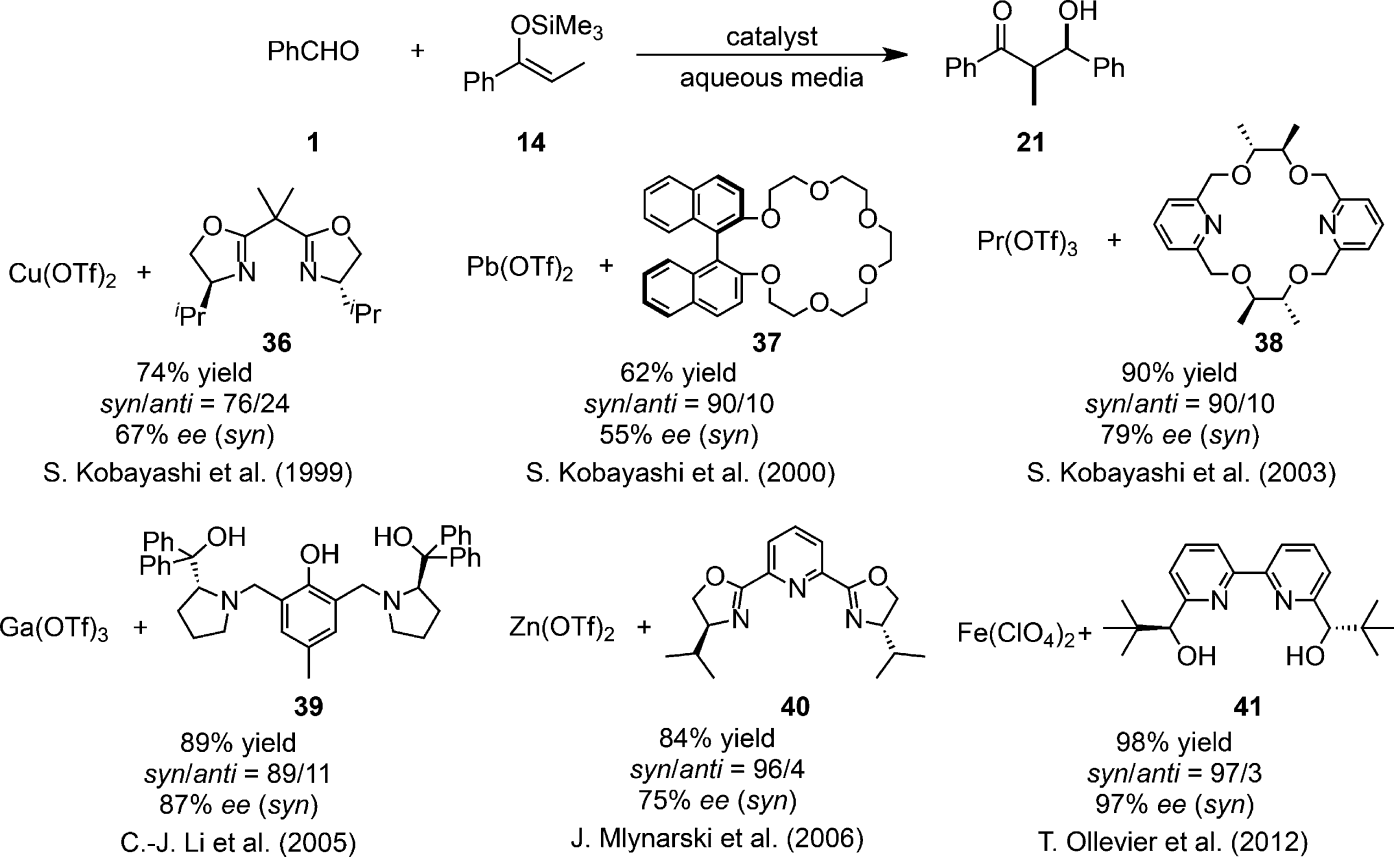
Catalytic asymmetric Mukaiyama aldol reaction between hydrophobic substrates in aqueous media.

Recent advances in Mukaiyama aldol reactions in aqueous media led chemists to examine the possibility of asymmetric catalysis in an aqueous environment. Almost all successful examples of catalytic asymmetric variants reported in organic solvents since 1990^92^ entailed absolutely aprotic anhydrous conditions as well as quite low reaction temperatures (e.g., −78 °C). Therefore the emergence of facile, convenient, and environmentally benign methodologies without tedious operations is desirable. However, a major difficulty in achieving asymmetric catalysis in aqueous media is the weakness of non-covalent interactions between substrates, chiral ligands, and metal ions under competitive polar conditions. Conversely, if it can be highly controlled, the construction of asymmetric environments in the presence of water is expected to inhibit undesired achiral side reactions and impose stricter stereochemical regulation than in organic solvents through the hydrogen bond network, specific solvation, and hydrophobic interactions. Despite the formidable task of controlling stereochemistry efficiently in an aqueous environment, recent attempts at this challenging endeavor have been successful. The successful catalyst systems for asymmetric Mukaiyama aldol reactions between hydrophobic substrates are shown in Scheme 20. The underlying strategies in common are: (i) the chiral aqua complex is stable; (ii) the replacement of the coordinated water molecule by an aldehyde is rapid and facile; and (iii) one enantioface of the coordinated aldehyde carbonyl is shielded effectively.

**Scheme 21:**
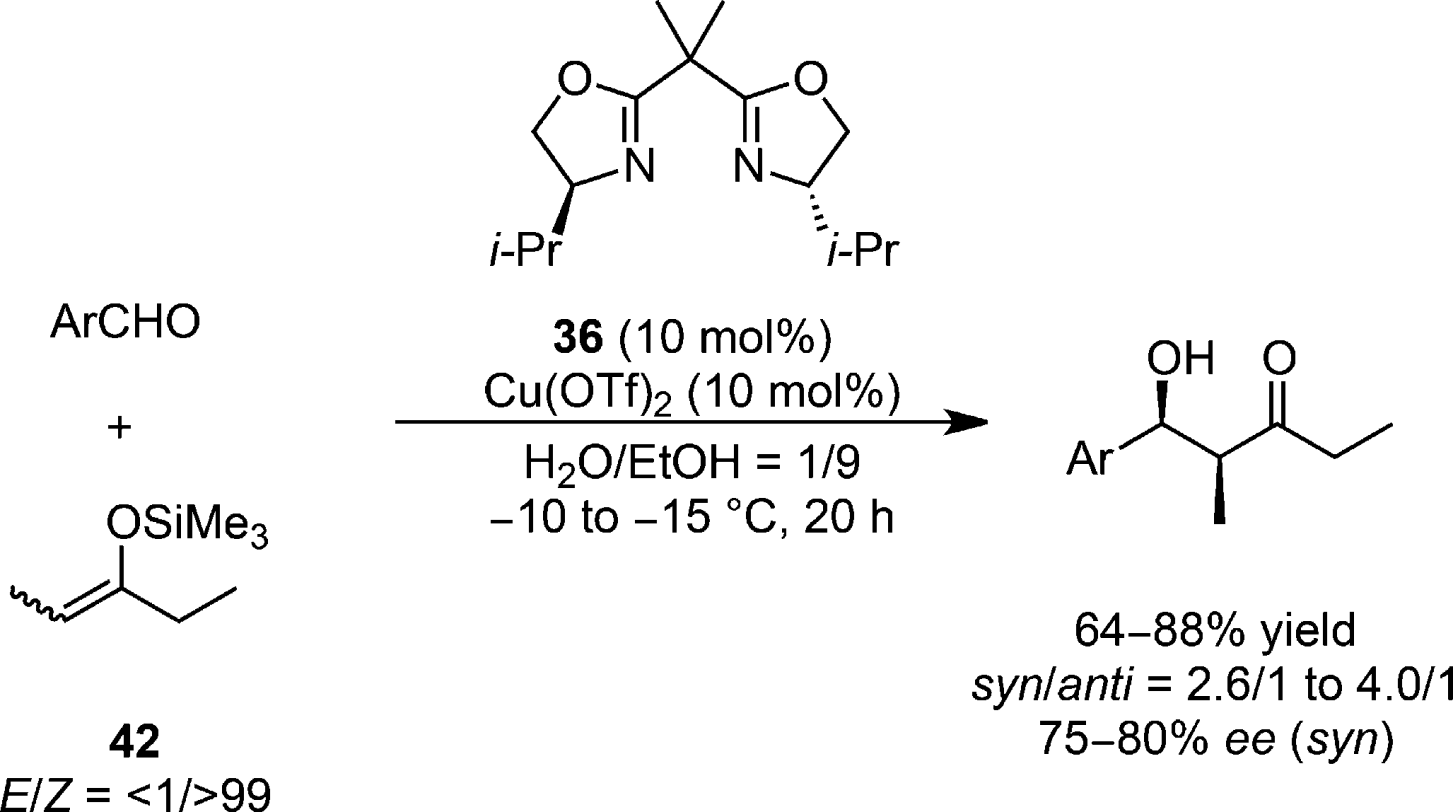
First example of an asymmetric Mukaiyama aldol reaction in aqueous media.

Copper(II) aqua structures were formed with chiral bis(oxazoline) templates, although these are normally prepared under anhydrous conditions.^93^,^94^ These results seemed to allude to the latent stability of the copper(II)-bis(oxazoline) complex **36** in an aqueous environment. Because of the well-known Lewis acid activity of copper(II) and its apparent stability in water, Kobayashi et al. examined copper(II)-bis(oxazoline) complexes as catalysts for asymmetric synthesis in aqueous media.^95^ The Mukaiyama aldol reactions of (*Z*)-enolate with aldehydes were catalyzed efficiently by a chiral copper(II) complex in aqueous ethanol (H_2_O:EtOH=1:9) to afford the desired aldol adducts in moderate to good yields and enantioselectivities (Scheme 21). It is noteworthy that much lower yields and selectivities were observed without water.

**Scheme 22:**
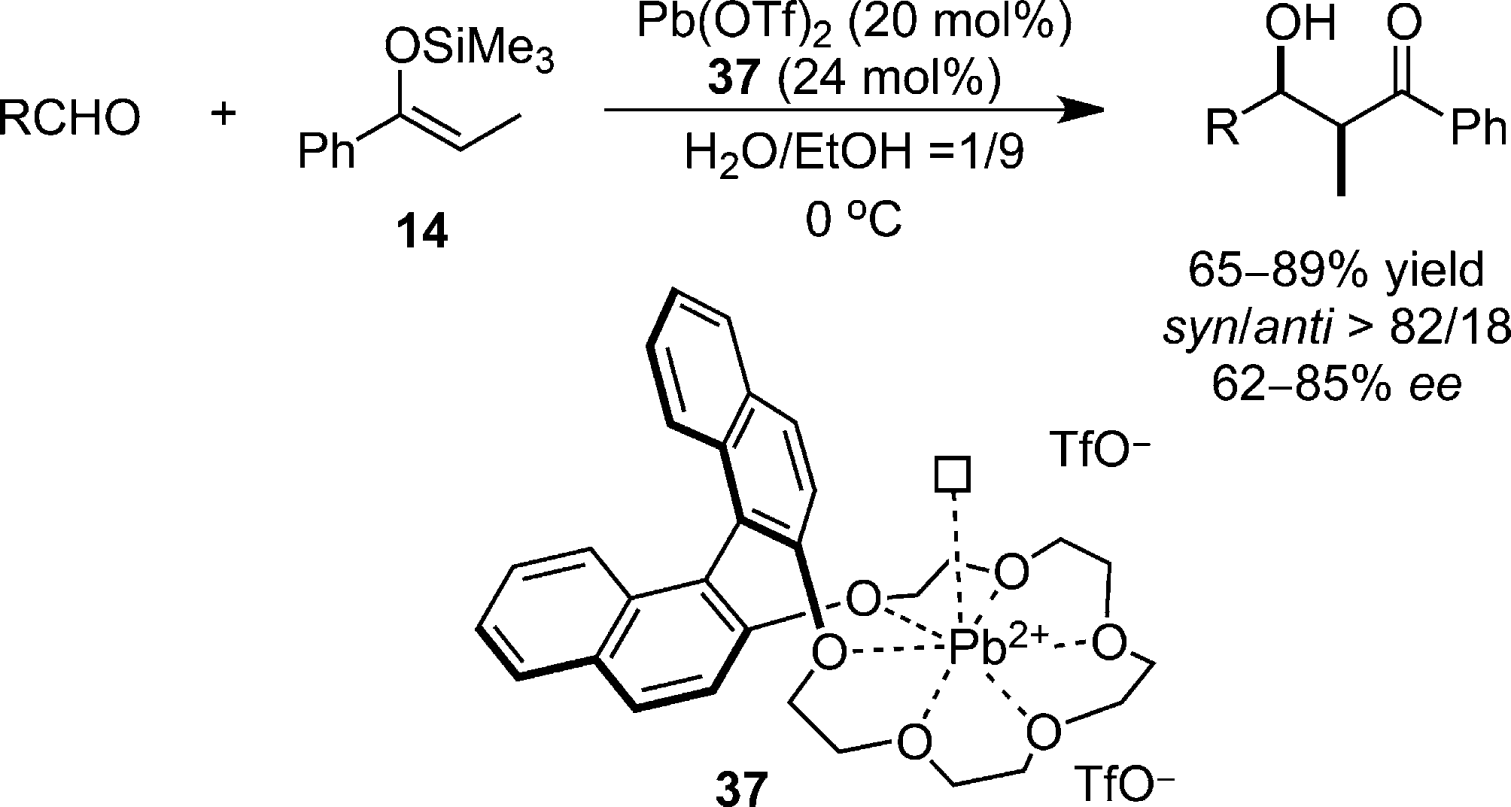
Chiral lead(II) catalyst for enantioselective Mukaiyama aldol reactions.

Exploitation of multicoordination systems is another possible solution for the instability of chiral metal complexes in aqueous environments. The examination of combinations of chiral crown ethers **37** with metal triflates on the basis of ionic radii^96^ and hole sizes^97^ led to the discovery of an efficient chiral lead(II) catalyst for asymmetric Mukaiyama aldol reactions in aqueous media (Scheme 22).^98^,^99^,^100^ Pb(OTf)_2_ and chiral crown ether **37** were quantitatively recovered by simple extraction. Furthermore, this system could be applied to thioester-derived silicon enolates as nucleophiles. A kinetic study performed for the chiral reaction and the Pb(OTf)_2_-catalyzed achiral reaction resulted in the observation of almost comparable reaction rates and the same levels of diastereoselectivity.

**Scheme 23:**
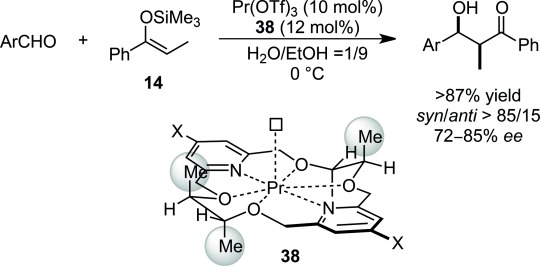
Chiral praseodymium catalyst for enantioselective Mukaiyama aldol reactions.

**Scheme 24:**
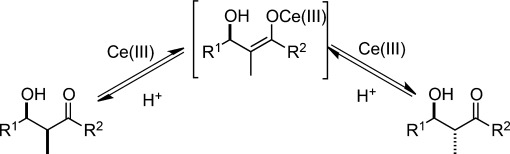
Plausible epimerization induced by lanthanides.

Inspired by these successes, intensive efforts have been devoted to stereoselective synthesis in aqueous environments and they have led to the emergence of highly efficient catalytic asymmetric Mukaiyama aldol reactions using water-compatible Lewis acids with chiral ligands. For example, the catalysts composed of lanthanide triflates and chiral bis-pyridino-18-crown-6 **38** exhibited higher diastereo- and enantioselectivity (Scheme 23).^45^,^101^,^102^ A vacant site as shown in Scheme 23 was suggested to be crucial for catalytic activity as a recipient of an aldehyde molecule. Because lanthanides are known to promote the epimerization between *syn*- and *anti*-adducts *via* keto-enolization, presumably because of their greater Lewis acidity in aqueous media (Scheme 24),^103^ multicoordination systems play a prominent role in chiral induction.

**Scheme 25:**
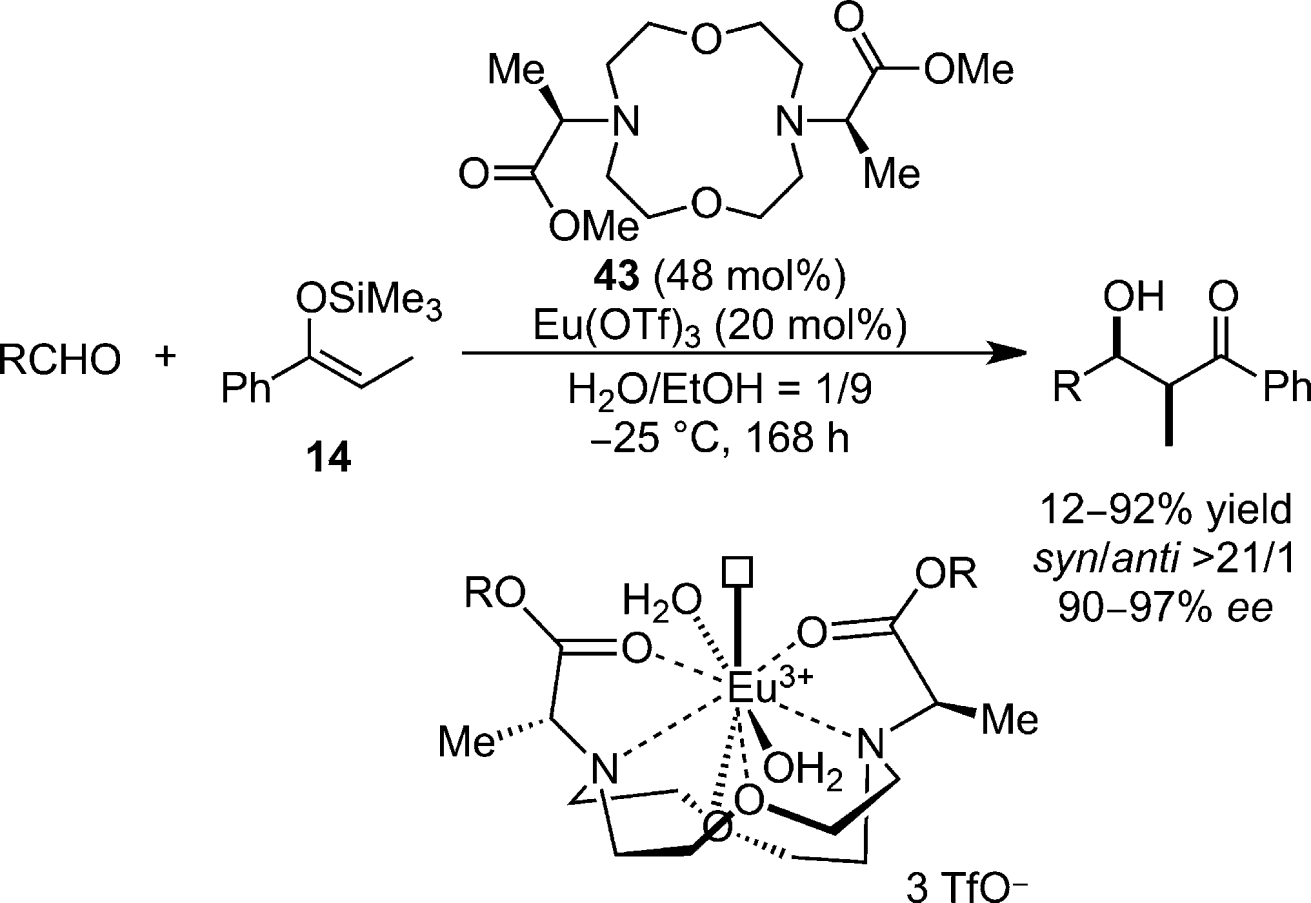
Chiral lanthanide catalysts for enantioselective Mukaiyama aldol reactions.

The fit between the crown ether and the metal cations is a prominent factor in attaining high selectivities; with the larger cations such as La, Ce, Pr and Nd providing the aldol adduct with high diastereo- and enantioselectivities, while the smaller cations such as Dy, Ho, Yb, Y and Sc resulted in lower selectivities. Introduction of an electron-donating (MeO) group at the 4-position of the pyridine rings led to higher selectivities for the larger cations and lower for the smaller cations. For the electron-withdrawing (Br) group, although a similar tendency was observed, the effect of ionic radii was more significant. The asymmetric reactions with lanthanide complexes had slightly decelerated reaction rates, despite the strong coordination of the Lewis base, compared with the corresponding achiral pathway without the chiral crown ether. To explore lanthanide catalysis,^104^ macrocyclic gadolinium-containing polyaminopolycarboxylate-based contrast agents for magnetic resonance imaging^105^ were modified, and Eu(III) or Nd(III) complexes were applied to asymmetric Mukaiyama aldol reactions in aqueous media (Scheme 25). Unfortunately these complexes possessed quite low catalytic activities and the activities were highly dependent on the substrate.^106^

**Scheme 26:**
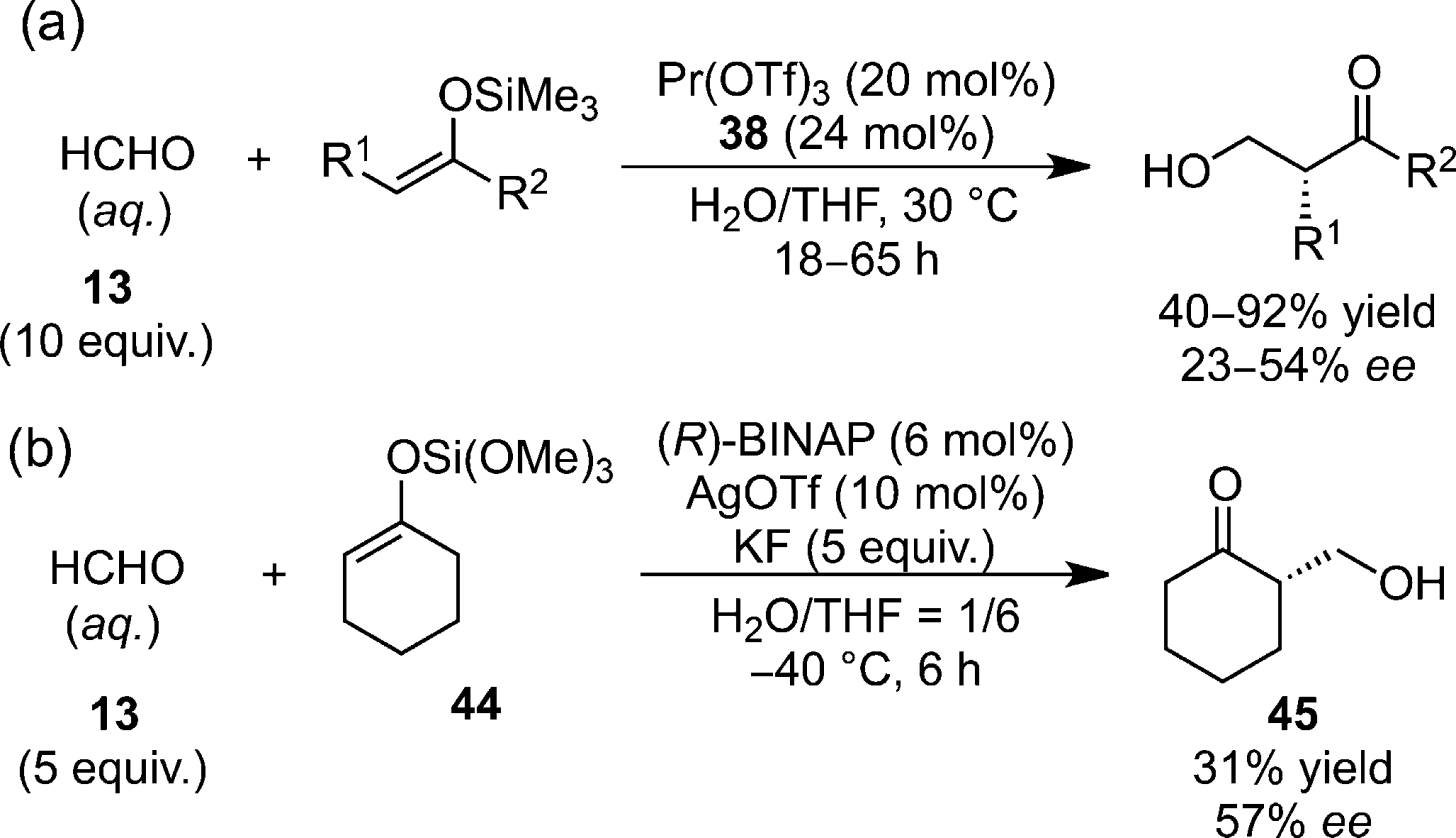
(a) Praseodymium- and (b) silver-catalyzed asymmetric hydroxymethylation in aqueous media.

The highly coordinative nature of these chiral crown ethers appears to be a key to understanding the catalytic behavior of lanthanides in an aqueous environment. In organic solvents, the coordination of heteroatoms generally leads to the loss of Lewis acidity of the metal, because of the behavior of the heteroatoms as Lewis bases. In contrast, it is known that subsequent coordination of water molecules generates a naked metal cation, which functions as a Lewis acid in an aqueous environment. A catalytic system comprised of Ln(OTf)_3_ and chiral bis-pyridino-18-crown-6 **38** in aqueous tetrahydrofuran was found to be effective for the asymmetric hydroxymethylation reaction (Scheme 26a).^107^ A bifunctional system of (*R*)-BINAP-AgOTf complex with a fluoride source was applied as a Lewis-base catalyst for the asymmetric hydroxymethylation of trimethoxysilyl enol ethers derived from cyclohexanone (31% yield, 57% *ee*) or α-tetralone (18% yield, 57% *ee*) in aqueous tetrahydrofuran, albeit in low yield (Scheme 26b).^108^

**Scheme 27:**
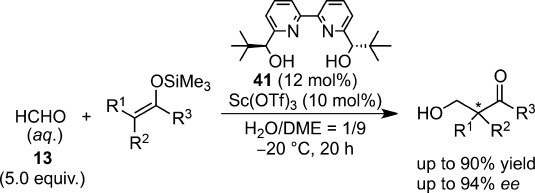
Scandium-catalyzed asymmetric hydroxymethylation in aqueous media.

In 2004, an excellent new catalytic system, based on a chiral scandium complex, was reported, with higher yields and enantioselectivities than previous systems (Scheme 27).^109^

**Scheme 28:**
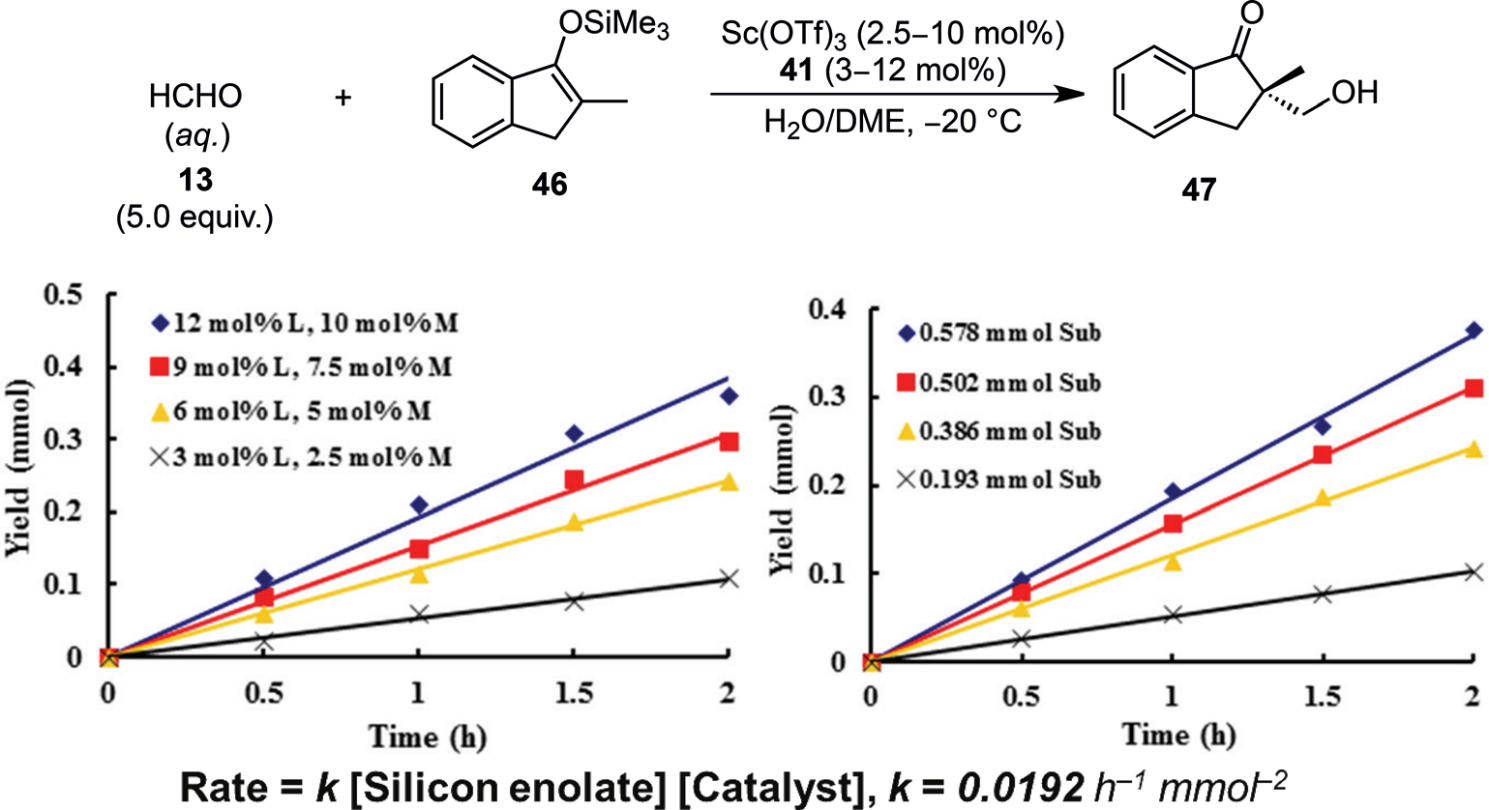
Mechanistic elucidation for scandium-catalyzed asymmetric hydroxymethylation in aqueous media. **L**=ligand **41**; **M**=Sc(OTf)_3_; **Sub**=substrate **46**.

It has been demonstrated for the first time that chiral 2,2′-bipyridine **41**^110^ is an exceedingly effective ligand for catalytic asymmetric reactions in an aqueous environment. Given its potential to chelate with many transition metal ions, the 2,2′-bipyridine backbone may represent a “privileged” ligand structure that can selectively catalyze many unique chemical reactions in an aqueous environment. The chiral scandium complex formed with **41** functioned most effectively in an H_2_O/DME solution. Under optimal conditions, asymmetric quaternary carbons can be constructed with high selectivities, and this methodology can be extended to various substrates such as thioester-derived silicon enolates. In some cases, competitive hydrolysis of silicon enolates occurred, but could be resolved by the addition of 2,6-di-*tert*-butylpyridine as a proton scavenger.^111^ Judging from X-ray analysis, the chiral scandium complex adopts a pentagonal bipyramidal structure where the chiral ligand is bound to the central scandium in a tetradentate manner. In a kinetic study,^112^ a first-order dependence on the silicon enolate **46** and the catalyst represents an overall rate law: *Rate*=*k*[Silicon Enolate][Catalyst]. The observation of the first-order dependence on both the silicon enolate and the catalyst negates the possible formation of a scandium enolate as an intermediate and the involvement of discrete molecules of the catalyst. Moreover, saturation kinetics was not observed, which also denies the direct bond formation between the chiral scandium catalyst and the silicon enolate (Scheme 28).

**Scheme 29:**
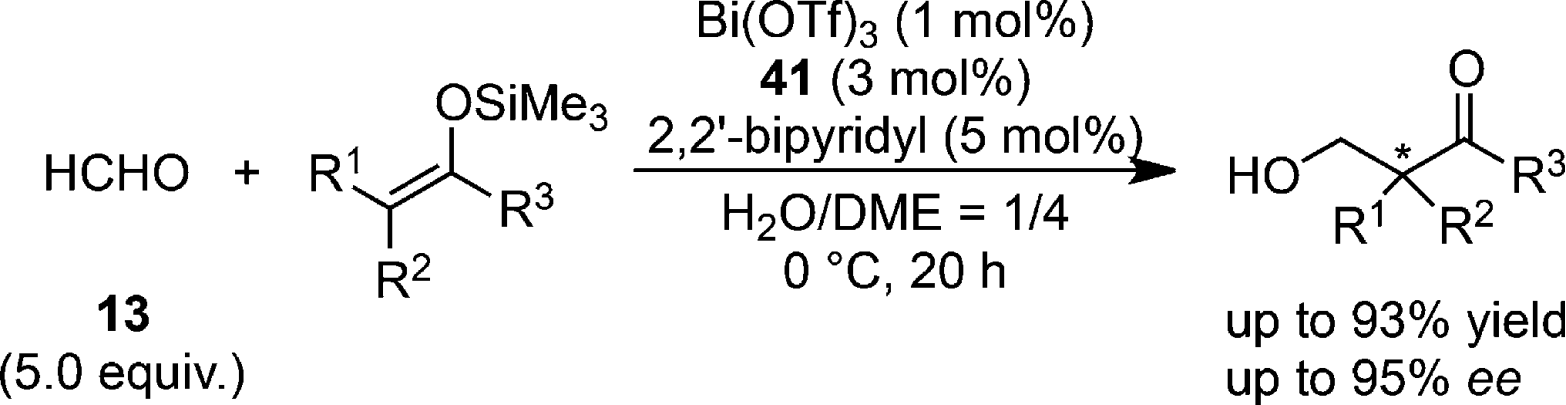
Bismuth-catalyzed asymmetric hydroxymethylation in aqueous media.

**Scheme 30:**
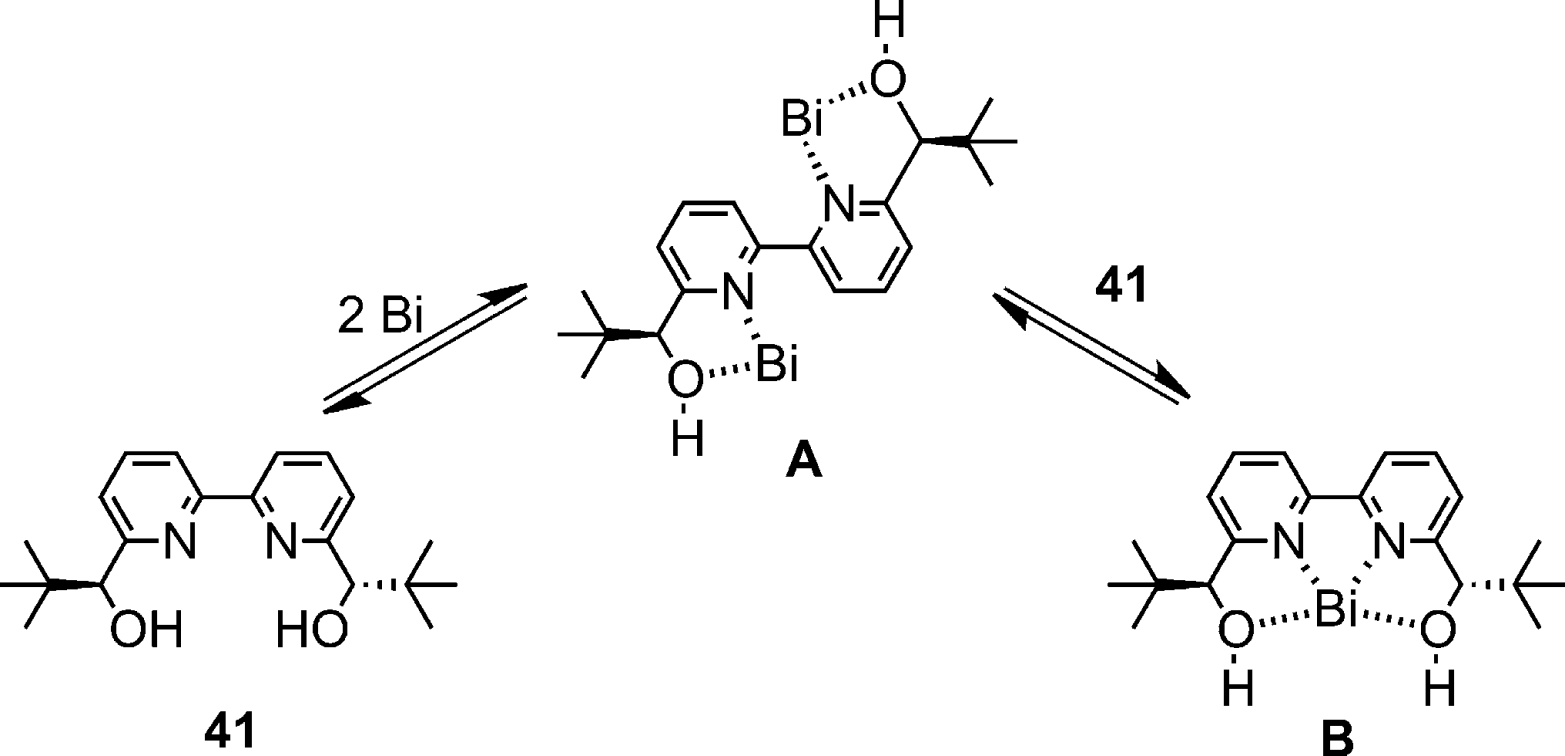
Equilibrium between bismuth-2,2′-bipyridine **41** complexes.

An extensive effort dedicated to asymmetric hydroxymethylation in aqueous media led to the discovery of a new catalytic system composed of Bi(OTf)_3_ and chiral 2,2′-bipyridine **41** (Scheme 29).^113^ Given the ease of hydrolysis in the presence of water,^114^ as well as the great discrepancy in the ionic diameters between bismuth (2.34 Å for 8-coordination) and scandium (1.74 Å for 8-coordination), this unexpected result offered interesting insight into asymmetric catalysis in an aqueous environment. Indeed, only a trace amount of hydroxymethylated adduct was obtained using Bi(OTf)_3_ in the absence of **41**, because of the rapid decomposition of the silicon enolate promoted by TfOH, which is generated readily from Bi(OTf)_3_ in water. The ligand acceleration effect of **41** suggests that the stabilization of Bi(OTf)_3_ occurs because of the coordination of **41** in water. A chiral bismuth catalyst consisting of 1 mol% of Bi(OTf)_3_, 3 mol% of **41**, and 5 mol% of 2,2′-bipyridyl was shown to afford the desired product in high yields with high enantioselectivity. Fundamental elucidation of the catalyst structure through NMR spectroscopy indicated that two equivalents of Bi(OTf)_3_ and one equivalent of **41** formed complex **A**, and that complex **B** consisting of one equivalent of Bi(OTf)_3_ and one equivalent of **41** was generated when an excess amount of **41** was added (Scheme 30).^114^ It is noted that complex **B** is stable even in the presence of 2,2′-bipyridine, and that **B** is readily formed from Bi(OTf)_3_-2,2′-bipyridine complex and **41**.

**Scheme 31:**
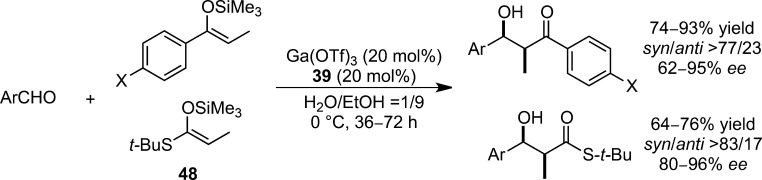
Chiral gallium catalyst for enantioselective Mukaiyama aldol reactions.

In 2002, Ga(OTf)_3_ with chiral Trost-type semicrown ligand **39**^115^ was discovered to be an efficient catalyst for asymmetric Mukaiyama aldol reactions in aqueous media (Scheme 31).^116^ UV-vis titration and ESI-MS analysis confirmed this gallium complex to be a 1:1 complex.^117^ Control experiments performed without the ligand suggested that it played a key role in accelerating the reaction and suppressing the hydrolysis of the silicon enolates. Comparably wide substrate scope was attained with satisfactory levels of diastereo- and enantioselectivities from substrates containing thioketene silyl acetals. However, the use of aliphatic aldehydes resulted in a significant loss of enantioselectivity.

**Scheme 32:**
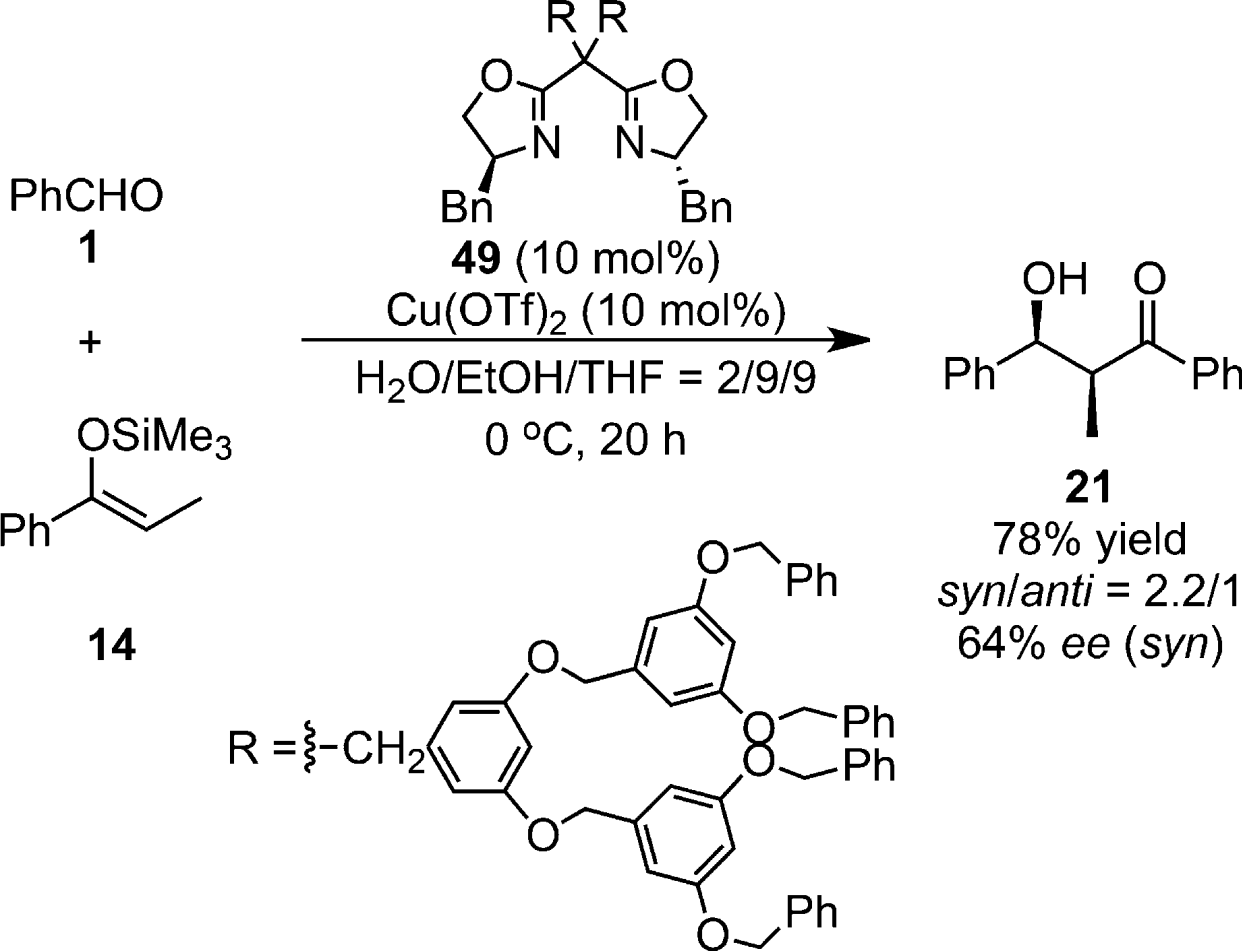
Chiral dendritic copper(II) catalyst for enantioselective Mukaiyama aldol reactions.

On the other hand, the *C*_2_-symmetric bis(oxazolines) **49** disubstituted with two Fréchet-type polyether dendrimers exhibited similar reactivities and enantioselectivities (up to 78% yield, *syn*:*anti*=2.2:1, 64% *ee* [*syn*]) for the asymmetric Cu(II)-catalyzed aldol reaction in aqueous media in comparison with Kobayashi’s previous work (98% yield, *syn*:*anti*=2.6:1, 61% *ee* [*syn*]) (Scheme 32).^118^

**Scheme 33:**
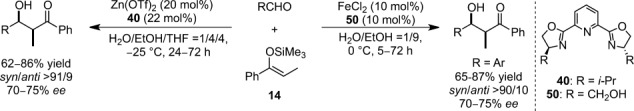
Chiral zinc(II) and iron(II) catalysts for asymmetric Mukaiyama aldol reactions.

Another promising approach focused upon the Lewis acidity of Zn(II) and Fe(II) to develop chiral catalysts formed with Zn(OTf)_2_ and *i-*Pr-*pybox* ligand **40**^119^ or FeCl_2_ and hydroxymethyl-*pybox* ligand **50**^120^ for the asymmetric Mukaiyama aldol reactions in aqueous media (Scheme 33). Although both aliphatic and aromatic aldehydes were applicable with moderate selectivity, aldehydes bearing heteroatoms and aqueous aldehydes were apt to be unsuccessful in the former catalytic system. Meanwhile, the latter catalyst was effective for the reactions of aromatic aldehydes.

**Scheme 34:**
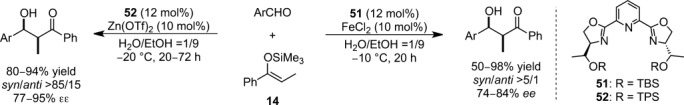
Designed chiral iron(II) and zinc(II) catalysts.

To overcome the instability, capriciousness, and sensitivity of chiral iron(II) and zinc(II) complexes to many reaction factors, tuned and lipophilic *pybox* ligands **51** and **52** were designed (Scheme 34).^121^ Although the selectivity improved comparatively in the reactions of some substrates, the reactions of aliphatic aldehydes still suffered from significant drops in reactivity and selectivity.

**Scheme 35:**
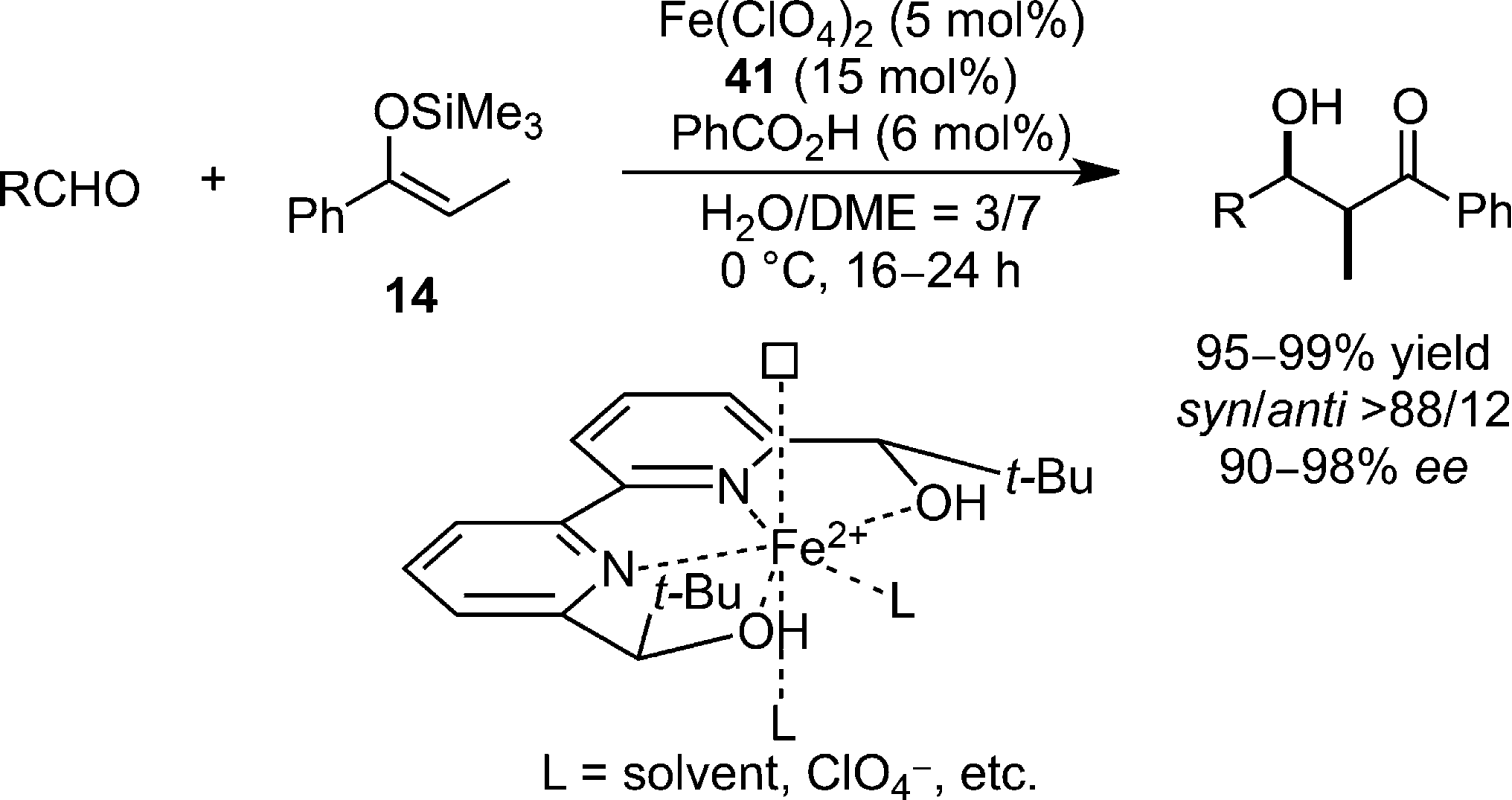
Highly efficient chiral iron(II) catalyst for enantioselective Mukaiyama aldol reactions.

In spite of these vigorous explorations pursuing efficient catalytic systems, there have still been insurmountable limitations in catalytic activity and substrate scope. Not only did almost all catalytic systems require 10–20 mol% of Lewis acids and 12–48 mol% of chiral ligands, but these methodologies possessed limited scope with some substrates, such as aliphatic aldehydes, for which a remarkable drop in enantioselectivity was commonly observed. In 2012, a wider substrate scope with outstanding stereoselectivity was achieved in the presence of a chiral iron(II) complex comprising Fe(ClO_4_)_2_ and chiral 2,2′-bipyridine ligand **41** (Scheme 35).^122^,^123^ Nevertheless, in this catalytic system, an excess amount of chiral ligand was required. Crystallographic investigations revealed that the central iron(II) ion is held in an almost square-planar fashion and the axial position of the pentagonal bipyramidal iron(II) complex is occupied by an additional water molecule. ESI-MS measurements corroborated the formation of a 1:1 complex in the reaction solution.^124^

Given the dominance of *Lewis acid-Lewis base interactions* over other interactions and the resulting loss of their acidity upon coordination to chiral ligands, Lewis acid-catalyzed asymmetric reactions in water using hydrophilic substrates are recognized as highly challenging, even though chiral induction can be achieved in aqueous media.^125^ The first example of Lewis acid-catalyzed asymmetric aldol reactions in pure water was reported as an application of LASC. As an extension of the chiral copper(II) catalyst in the water-ethanol system, the combination of Cu(DS)_2_ and chiral bis(oxazoline) **36** was employed.^126^ The LASC/Brønsted acid system mentioned above was further extended to this asymmetric reaction (Table 6).^127^,^128^ Benzoic acid and lauric acid as additives were effective not only for reactivity but also for enantioselectivity (up to *syn*:*anti*=2.8:1, 69% *ee* [*syn*]). Neither a strong Brønsted acid (CSA) nor a weaker one (phenol) could improve the yield and the selectivity. Even with a stoichiometric amount of the scandium salt, the Brønsted acid was also found to accelerate the reaction. This implied that the rate-determining step accelerated by the Brønsted acid was not the catalyst turnover step, but the nucleophilic addition step.^129^,^130^

**Scheme 36:**
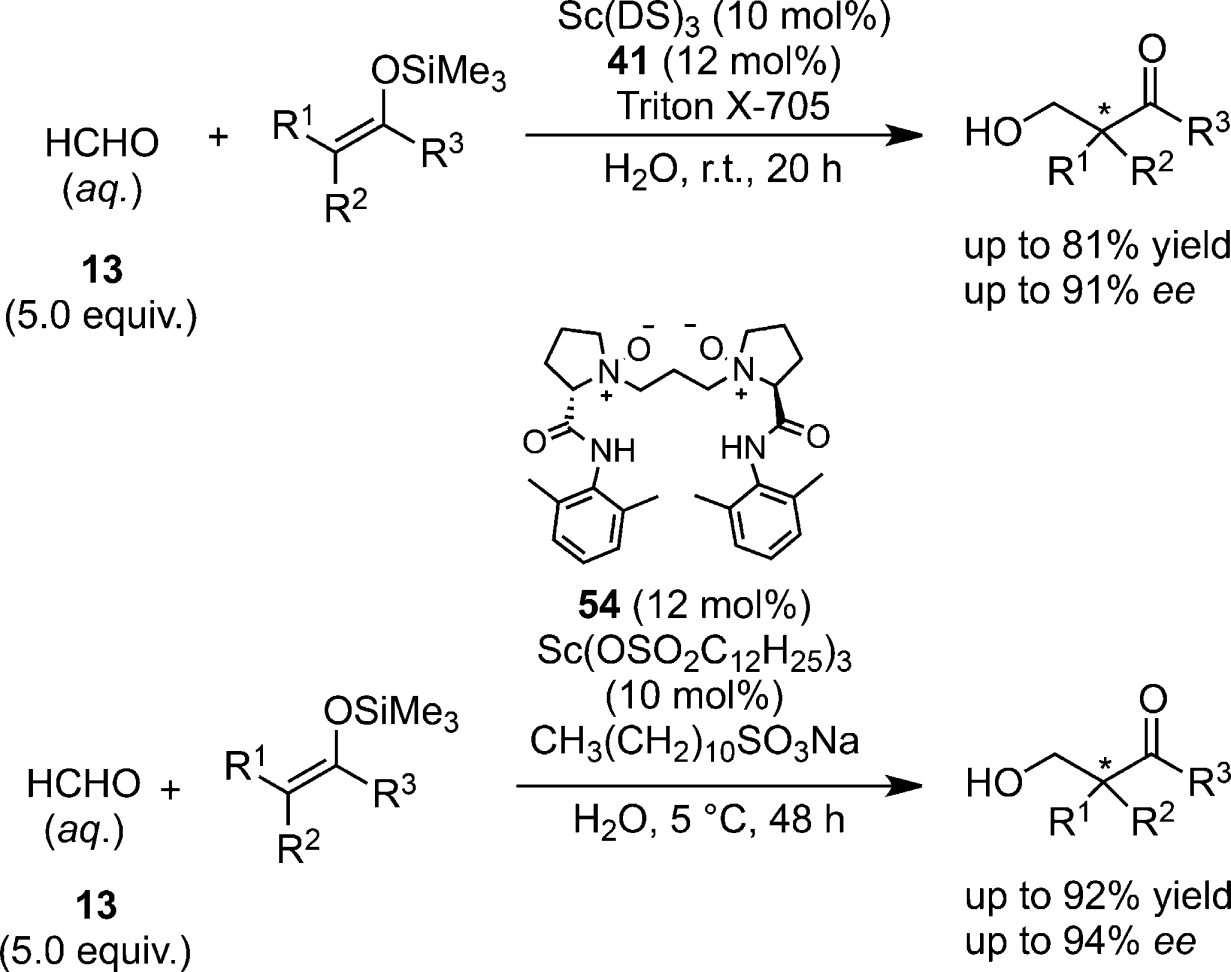
Asymmetric Mukaiyama aldol reactions using formaldehyde in water.

Catalytic asymmetric hydroxymethylation reactions were successfully carried out with 10 mol% of Sc(DS)_3_ and 12 mol% of chiral 2,2′-bipyridine **41** in the presence of Triton X-705 or with 10 mol% of Sc(OSO_2_C_12_H_25_)_3_ and 12 mol% of chiral *N*-oxide ligand **54**^131^ in the presence of C_12_H_25_SO_3_Na to afford the desired aldol adducts in high yields with high enantioselectivities (Scheme 36).^132^ A wide range of silicon enolates including thioketene silyl acetals reacted smoothly and high enantioselectivities were attained. Centrifugation of the reaction mixture (3000 rpm, 20 min) led to the successful separation of the colloidal dispersion into three phases; the upper, middle, and bottom phases corresponding to the water, surfactant, and organic layers, respectively.

**Scheme 37:**
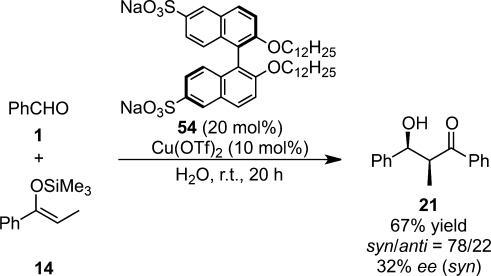
Chiral surfactant for enantioselective Mukaiyama aldol reactions.

Chiral disulfonated binaphthyl dialkyl ethers with Ga(OTf)_3_ and Cu(OTf)_2_ catalyzed the asymmetric Mukaiyama aldol reactions more efficiently than did Sc(OTf)_3_ in water with moderate selectivities (Scheme 37).^133^ The chiral amphiphile, which has been mainly used in enantiomer separation by capillary electrochromatography,^134^ formed micelles in water and the changes in the torsion between two naphthyl rings might result in chiral induction.

**Scheme 38:**
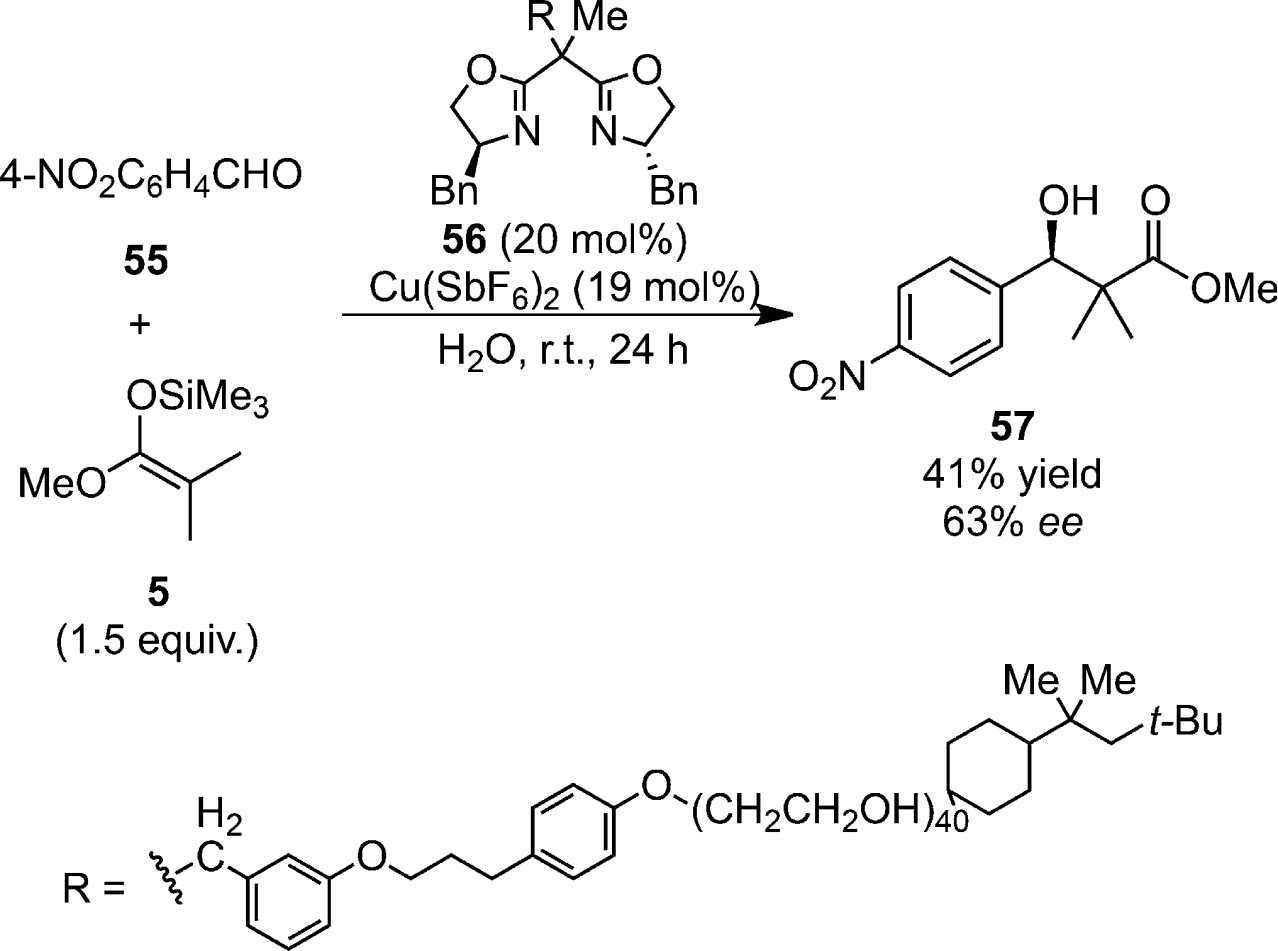
Triton®-supported chiral copper(II) complex for enantioselective Mukaiyama aldol reactions.

PEG- or Triton®-supported chiral bis(oxazoline) has been employed in combination with copper(II) salts as a catalyst for the Mukaiyama aldol reactions of ketene silyl acetal derived from methyl isobutyrate with aldehydes in water (Scheme 38).^135^

**Scheme 39:**
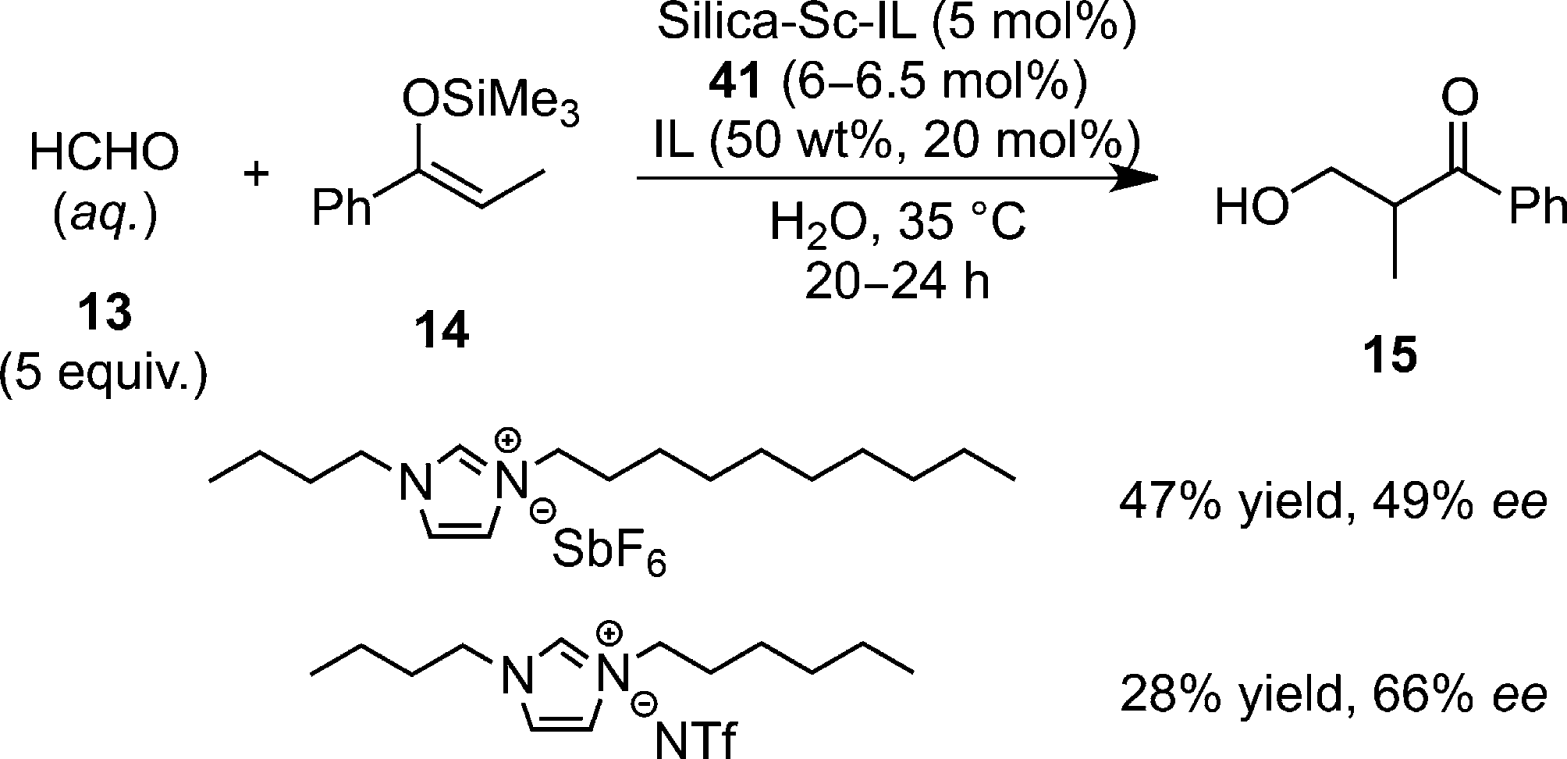
Catalytic asymmetric hydroxymethylation using Silica-Sc-IL.

The Silica-Sc-IL system was also rendered asymmetric by the addition of **56** (up to 28% yield, 66% *ee*, Scheme 39).^136^ The high solubility of these ligands in water allowed a very convenient catalyst-recycling procedure.

## 7 Conclusions and Perspective

Mukaiyama aldol reactions in aqueous media have been surveyed. While the original Mukaiyama aldol reactions were carried out using stoichiometric amounts of Lewis acids such as TiCl_4_ in organic solvents under strictly anhydrous conditions, the reactions were also found to proceed in water. The first discovery was just the use of water in the reactions, which might work as a Lewis base. However, the reactions proceeded slowly and the yields were moderate. It was then discovered that Mukaiyama aldol reactions proceeded smoothly in the presence of lanthanide triflates in aqueous solvents. A catalytic amount of lanthanide triflates worked well and high yields with wide substrate scope were obtained. The original Mukaiyama aldol reactions were expanded significantly, because the reactions proceeded under milder conditions and strict anhydrous conditions were not required. Moreover, water-soluble substrates, water-containing substrates, and substrates bearing basic nitrogen atoms and free hydroxy groups and others can be used in aqueous Mukaiyama aldol reactions. The catalyst, lanthanide triflate, can be easily recovered and reused, and the reactions are thus suitable for green sustainable chemistry.

Another important aspect of this discovery of the lanthanide-catalyzed aqueous Mukaiyama aldol reactions is the expansion of the concepts of Lewis acids and organic reactions in water. Before this discovery, it was believed that Lewis acids decomposed rapidly even in the presence of small amounts of water. However, it was found that lanthanide triflates worked well as catalysts under aqueous conditions, and this finding led to the discovery of a series of water-compatible Lewis acids. Scandium triflate is now one of the most popular and frequently used Lewis acids in organic synthesis; it was discovered during this work as a water-compatible Lewis acid. Water-compatible Lewis acids have evolved to water-compatible chiral Lewis acids for asymmetric Mukaiyama aldol reactions. Several new concepts have been introduced to stabilize chiral Lewis acids under aqueous conditions, and these have been applied to other asymmetric reactions in aqueous media.

On the other hand, progress has also been made with organic reactions in water. Water is a clean, non-toxic, and inexpensive solvent and thus is suitable for green sustainable chemistry. In addition, many excellent *in vivo* reactions are carried out using enzymes as catalysts under aqueous conditions. Aqueous environments often produce singular phenomena not observed in conventional organic solvents, including rate acceleration and different selectivities. These beneficial effects in the presence of water are ascribed to its high dielectric constant and high cohesive energy density. The favorable transition states are stipulated by entropy-driven aggregation, dipole-dipole interaction, rapid proton-transfer in the order of picoseconds and so on.^137^ Dissociation is sometimes highly accelerated by the nucleophilic addition of water.

The chemistry in this field is, however, still immature because of an intimate dependence of catalyst structure on substrates. Compared with the well-refined catalytic systems for asymmetric hydroxymethylation in aqueous media, contemporary catalysts for reactions between hydrophobic substrates have the fault of substrate specificity and lack broader catalytic activities. The advances reported here will have an immense impact on organic chemistry as an expedient, environmentally benign and highly efficient tactic capable of accessing optically active aldols. Moreover, the role of water in Mukaiyama aldol reactions warrants further investigations.
